# Finite Element Implementation of SAFE for Asymmetric Stream‐Aquifer Flow Exchange

**DOI:** 10.1111/gwat.70098

**Published:** 2026-08-15

**Authors:** G. Kourakos, H. Morel‐Seytoux, E. C. Dogrul, T. Kadir, H. E. Dahlke

**Affiliations:** ^1^ Department of Land, Air and Water Resources University of California, Davis 1 Shields Avenue Davis CA 95616; ^2^ Hydroprose Consulting International Santa Rosa CA 95409; ^3^ California Department of Water Resources 1516 9th Street Sacramento CA 95814

## Abstract

Climate change and human activities increasingly affect interconnected stream–aquifer systems, motivating continued evaluation of how stream–aquifer interactions are represented in integrated groundwater–surface water models. Most groundwater models quantify stream–aquifer exchange using an empirical riverbed leakance parameter that is predefined or calibrated from observed head differences between the stream and aquifer. Here, we describe the numerical implementation of the analytical stream aquifer flow exchange (SAFE) formulation (Morel‐Seytoux et al., 2018) within the finite‐element‐based Integrated Water Flow Model (IWFM). SAFE represents stream–aquifer exchange using physically based parameters and provides an alternative to commonly used first order exchange (FOE) formulations, such as the MODFLOW River Package (RIV). A key extension is the ability to quantify asymmetric stream–aquifer exchange arising from different groundwater heads on opposing sides of a stream, a capability not available in standard stream–aquifer interaction packages. Numerical experiments using hypothetical test cases and a large‐scale regional application demonstrate that differences in conductance and connectivity parameterizations produce distinct spatial and temporal patterns of simulated exchange. Compared with FOE, the SAFE formulation produces smoother temporal transitions and reduced short‐term variability in simulated exchange. These results demonstrate the importance of conceptual model formulation when interpreting simulated stream–aquifer exchange and provide a physically based alternative for large‐scale integrated groundwater–surface water modeling.

## Introduction

The interaction between streams and groundwater is a significant process in the hydrological cycle. An improved representation and quantification of stream–aquifer interaction are crucial for water management (Khan and Khan 2019) such as the conjunctive use of surface and groundwater (McCallum et al. 2013; Dogrul et al. 2016; Kerebih and Keshari 2021), water rights allocation (Cech 2010; Condon and Maxwell 2013), and riverine ecosystem management (Smith et al. 2008; Sonne et al. 2018) to name a few. Several studies (Sophocleous 2002; Brunner et al. 2017; Haque et al. 2021) provide comprehensive reviews on recent advances to understand and quantify the flow exchange between streams and groundwater.

Many local‐scale research studies have focused on biological, chemical, and physical processes influencing flow, sediment transport, stream–aquifer interaction, and water quality in stream‐groundwater systems (Song et al. 2007; Boano et al. 2014; Ulrich et al. 2015; Constantz 2016). These studies have revealed that biofilm growth (Shrivastava et al. 2020) and the deposition of fine sediments can lead to streambed clogging and reduced flow exchange between the stream and the aquifer (Datry et al. 2015; Naganna et al. 2017). At the regional scale, studies have investigated the role that stream–aquifer interaction has on contaminant transport (Kalbus et al. 2007; Lapworth et al. 2012) as well as the impact of stream alterations or groundwater pumping on hyporheic exchange (Boano et al. 2014).

Although great advances have been made to increase mechanistic understanding of flow processes between rivers and underlying aquifers and their controlling factors, most integrated surface water–groundwater models represent stream–aquifer exchange using simplified parameterizations that are computationally efficient and well suited for large‐scale applications. Most groundwater models estimate the flow exchange between a stream channel and the underlying aquifer based on Darcy's law by calibrating a “leakance” factor which determines the conductivity of a clogging layer within the riverbed divided by its layer thickness, and by considering the head difference between the river and the groundwater table just below the streambed bottom (McDonald and Harbaugh 1988). Among the available numerical codes, MODFLOW (Harbaug et al. 2017) is the most used tool to simulate stream–aquifer interaction using the finite difference numerical scheme. MODFLOW provides several packages such as RIV (Harbaugh 2005), STR (Prudic 1989), or SFR2 (Niswonger and Prudic 2005) to estimate the flow exchange between a stream and groundwater. In addition to finite difference models, there also exist several finite‐element‐based numerical codes for groundwater flow modeling such as HydroGeoSphere (Therrien et al. 1996; Aquanty Inc. 2015), IWFM (Dogrul and Kadir 2023). These models are also frequently used in large‐scale integrated modeling (Hassan et al. 2014; Tian et al. 2015; Kourakos et al. 2019; Boubacar et al. 2020; Faunt et al. 2024).

Most models approximate the stream–aquifer exchange as a predominantly vertical flow process based on the head difference between the stream and the groundwater at the stream node/cell multiplied by a leakance coefficient. There are several issues with this approach, as discussed by Morel‐Seytoux et al. (2018) who concluded that the leakance coefficient “is a very complex parameter that depends on other unknown parameters.” Most importantly, Morel‐Seytoux et al. (2018) pointed out that the leakance coefficient is an effective parameter that aggregates several physical processes and properties into a single term similar to, for example, hydraulic conductivity or specific yield, and it is usually estimated via calibration. However, the value of leakance depends on the hydrological conditions, which may change over time; therefore a single coefficient may not be applicable for a long period or for the simulation of system changes that exceed historical observations.

To address some of the limitations associated with fixed leakance parameterizations, Rushton (2007) proposed a river coefficient that is a function of horizontal conductivity times a factor and provided detailed numerical examples to assess suitable values for the proposed factor. Morel‐Seytoux and Daly (1975) developed the concept of reach transmissivity, which was later extended by Morel‐Seytoux (2009) into the stream aquifer flow exchange (SAFE) method. The SAFE method is an analytical framework in which an integrated physical conductance coefficient called SAFE is calculated as a function of several physical parameters (e.g., wetted perimeter, stream head, etc.). The method was developed for channels with an elliptical cross‐section until Morel‐Seytoux et al. (2014, 2017) broadened the approach for use in different systems by supporting customizable factors such as normalized wetted perimeter, degree of penetration of the river, presence of a clogging layer, and anisotropic aquifer hydraulic conductivity.

In large‐scale modeling the stream–groundwater interaction can assume three different states: (1) gaining stream, when the groundwater head is higher than the stream head and the stream receives aquifer return flow (RF), (2) losing connected stream, when the groundwater head drops below the stream head and the stream discharges to the groundwater aquifer, and (3) losing disconnected stream, when the groundwater head falls below a certain threshold and the stream gets disconnected from the groundwater. For the first and second states, the interaction is proportional to the stream groundwater head difference. For the third state, the infiltration rate becomes constant as it is independent of the groundwater head; instead, it does depend on the disconnection elevation. In MODFLOW, IWFM, and other software packages, a common approach is to assume that the disconnection occurs when the groundwater head drops below the bottom of the riverbed. This assumption represents a pragmatic numerical criterion rather than a physical discontinuity, and has proven effective in many applied studies. In practice, there is a transition zone between connected and disconnected states where the dependency of the infiltration rate on the groundwater head gradually decreases. This issue has been discussed by Osman and Bruen (2002), Brunner et al. (2009, 2011), McMahon and Nathan (2021), who concluded that this simple approach underestimates the actual infiltration rate. Brunner et al. (2009) also showed that a simple disconnection criterion, for example, the stream stage is twice the stream width (Sophocleous 2002), is not correct. Furthermore, Brunner et al. (2009) developed a mathematical formula that captures the premise that a groundwater mound can form under streams which can maintain the connection between the stream and groundwater for long periods of time. The formula that determines when the stream gets disconnected depends on the leakance coefficient among other parameters. Following a similar approach, Morel‐Seytoux et al. (2016) developed an analytical formulation to express the disconnection criterion as a function of the dimensionless SAFE parameter, aquifer hydraulic conductivity, entry pressure and other parameters.

Most large‐scale stream–aquifer interaction approaches calculate a flow exchange volume between the stream and the aquifer with the assumption that the groundwater head is symmetric on the left and right side of the stream. Because rivers commonly coincide with political or administrative boundaries, groundwater conditions on opposite banks may diverge where groundwater development and management differ across jurisdictions (Rodriguez et al. 2020; Sterckx et al. 2026). Capturing and representing these head differences in numerical models can be critical as they might differentially impact the flow exchange between the river and groundwater, particularly if streams are large and wide (Feitelson 2000). Using highly detailed numerical models, Texier et al. (2022) show that differential pumping (e.g., pumping on just one side of the stream) can result in asymmetric stream–aquifer discharges. Balbarini et al. (2017) also showed that the shape of the river and its relationship with the regional groundwater flow results in asymmetric flow exchange between stream and aquifer. Nevertheless, these works used highly detailed numerical models to quantify the asymmetric flow exchange because of different groundwater heads on either side of the stream. While these studies demonstrate the importance of asymmetry, their computational demands limit their applicability in regional‐scale integrated modeling. Nemeth et al. (2000) and Nemeth and Solo‐Gabriele (2003) applied the reach transmissivity method (Morel‐Seytoux and Daly 1975) to estimate asymmetric leakage beneath a levee in Florida. Miracapillo and Morel‐Seytoux et al. (2014) generalized the analytical solutions for the symmetric case (Morel‐Seytoux 2009; Morel‐Seytoux et al. 2014) to calculate different discharges on the right and left side of the stream, which makes the approach more suitable for large‐scale modeling applications.

The works by Morel‐Seytoux and others have advanced the theoretical foundation of the SAFE method. However, their applications are generally restricted to simplified, hypothetical examples (2D cross‐sections). The primary objective of this study is to present and document the numerical implementation of the existing analytical SAFE formulation within a finite‐element‐based integrated groundwater–surface water model (IWFM). A second objective is to examine how this physically based parameterization influences the numerical behavior of simulated stream–aquifer exchange relative to a commonly used conductance‐based first order exchange (FOE) approach, using both hypothetical and regional‐scale case studies. Finally, this work demonstrates a novel finite‐element implementation of asymmetric stream–aquifer exchange that resolves left‐ and right‐bank contributions under spatially variable groundwater head conditions—an ability that is absent from most stream–aquifer interaction packages.

These objectives are intentionally focused on numerical implementation, formulation, and model behavior rather than predictive accuracy. Accordingly, this study evaluates the implications of alternative process representations for simulated stream–aquifer exchange within a large‐scale modeling framework, rather than validating model outputs against field observations or analytical benchmarks. A rigorous validation would require development of dedicated benchmark problems or site‐specific applications, independent observation data, and full recalibration of each formulation, which is beyond the scope of the present numerical implementation study. The comparisons presented here are therefore designed to identify differences in model behavior arising from the underlying formulations rather than to assess the absolute predictive performance. Such validation is beyond the scope of this implementation‐focused work and is reserved for future applied studies.

The remainder of this paper is organized as follows. The Methods section describes the finite‐element implementation of the SAFE formulation for saturated stream–aquifer connection and its extension to asymmetric groundwater head conditions on either side of the stream. The Results section presents numerical applications to both a hypothetical system and a regional‐scale basin, followed by a Discussion and Conclusion that synthesize the implications of the findings for large‐scale integrated groundwater–surface water modeling.

## Methods

### Governing Groundwater Flow Equation and Stream–Aquifer Coupling in IWFM

IWFM simulates 3D transient groundwater flow in a multilayer aquifer system using a Galerkin finite‐element formulation of the groundwater mass conservation equation. Aquifer layers may be confined, unconfined, or leaky, and may be separated by aquitards or aquicludes. Groundwater heads are solved simultaneously with stream and lake flows, allowing dynamic feedback between surface water and groundwater components (Dogrul 2025). For each aquifer layer, groundwater flow is governed by the conservation of mass expressed as:

(1) $$\begin{array}{ll} S_{s}\frac{\partial h}{\partial t}\nabla \cdot q & =q_{u}+q_{d}+\delta \left(x-x_{s},y-y_{s}\right)\frac{Q_{s\mathrm{int}}}{A_{s}}\\ & \;+\delta \left(x-x_{\mathrm{lk}},y-y_{\mathrm{lk}}\right)\frac{Q_{\mathrm{lk}\mathrm{int}}}{A_{\mathrm{lk}}}\\ & \;+\delta \left(x-x_{\mathrm{td}},y-y_{\mathrm{td}}\right)\frac{Q_{\mathrm{td}}}{A_{\mathrm{td}}}-q_{\mathrm{et}}-q_{\mathrm{rt}} \end{array}$$

where *h* is groundwater head, *S*_*s*_ is storativity (or specific yield for unconfined layers), *q* is the Darcy flux vector, *q*_*u*_ and *q*_*d*_ represent vertical flow exchange with overlying and underlying layers, and *q*_*et*_ and *q*_*rt*_ denote evapotranspiration losses and groundwater RF, respectively. The Dirac delta functions localize source–sink terms associated with stream–aquifer interaction (*Q*_*s*int_), lake–aquifer interaction (*Q*_*lk*int_), and tile drainage or subsurface irrigation (*Q*_*td*_) at their respective spatial locations, normalized by effective interaction areas (Dogrul 2025).

In IWFM, the groundwater flow equation is discretized spatially using linear triangular or bilinear quadrilateral finite elements and integrated in time using an implicit scheme. All hydrologic components, including groundwater, streams, and lakes, are assembled into a single system of equations and solved simultaneously at each time step, ensuring mass conservation and consistent coupling among components (Dogrul 2025). The SAFE implementation modifies only the formulation of stream–aquifer exchange fluxes (*Q*_*s*int_) and does not alter the governing groundwater flow equation, numerical discretization, or solution strategy employed by IWFM.

### Stream–Aquifer Interaction Formulations

We first describe the conductance‐based approach commonly implemented in IWFM, MODFLOW, and related models. We then present the analytical SAFE formulation developed by Morel‐Seytoux et al. (2014, 2016, 2017, 2018, 2019), followed by its extension to account for asymmetric groundwater heads on the left and right sides of the stream and the resulting implications for flow exchange.

#### FOE Stream–Aquifer Interaction

One of the most common approaches used to simulate river‐aquifer seepage in integrated surface water–groundwater models is the River Package (RIV) of MODFLOW (Harbaugh 2005). In this approach, stream–aquifer exchange is calculated as follows:

(2) $$Q_{s}=\frac{K_{\mathrm{cl}}}{e_{\mathrm{cl}}}LW_{\mathrm{per}}\left(h_{s}-h_{\mathrm{gw}}\right)$$

where $K_{\mathrm{cl}}$ is the riverbed hydraulic conductivity, $e_{\mathrm{cl}}$ is riverbed thickness, L is the stream segment length that is associated with a given stream node, $W_{\mathrm{per}}$ is the wetted perimeter on the same stream node, $h_{s}$ is the stream head, and $h_{\mathrm{gw}}$ is the groundwater head.

When $h_{s}>h_{\mathrm{gw}}$, seepage discharge from the stream to the aquifer occurs; when $h_{\mathrm{gw}}>h_{s}$, groundwater return flow (RF) to the stream occurs. In practice, the head difference is evaluated as $h_{s}-\max\left(h_{\mathrm{gw}},h_{\text{rbed}}\right)$, where $h_{\text{rbed}}$ is the elevation of the riverbed bottom. When groundwater head falls below $h_{\text{rbed}}$, the stream is assumed to be hydraulically disconnected, and seepage discharge becomes independent of groundwater head and proportional to stream stage alone. Variants of this formulation are implemented in models such as MODFLOW (McDonald and Harbaugh 1988) and IWFM (Dogrul, 2011). Collectively, these approaches are referred to here as first‐order exchange (FOE)‐type methods, as the rate of water exchange is linearly proportional to the head difference between the stream and the aquifer.

It should be noted that some integrated models, including HydroGeoSphere (Therrien et al. 1996; Aquanty Inc. 2015) and ParFlow (Maxwell et al. 2009), also provide more advanced formulations in which hydraulic head (pressure) is continuous across the streambed interface through a fully coupled surface–subsurface solution. These approaches eliminate the need to prescribe conductance and allow exchange to be calculated directly from the governing equations. However, such formulations are typically more computationally demanding and can exhibit numerical instability in the unsaturated zone, which presents practical challenges for large‐scale regional applications. The focus of this work is on methods suitable for large‐scale regional applications, where FOE‐type and related reduced‐order formulations remain practical and widely adopted.

#### SAFE Formulation

In the SAFE formulation, stream‐aquifer exchange is expressed as a function of a physically derived conductance (i.e., the dimensionless SAFE coefficient) and the head difference between the stream and the aquifer:

(3) $$Q_{s}=2L\Gamma K_{H}\left(h_{s}-\max\left(h_{\mathrm{gw}},h_{\text{incip}}\right)\right)$$

where $K_{H}$ is the horizontal hydraulic conductivity of the aquifer, Γ is a dimensionless conductance coefficient representing the integrated flow resistance between the stream and the aquifer (Morel‐Seytoux 2009), $h_{\text{incip}}$ is the incipient desaturation head that defines the onset of the hydraulic disconnection (Morel‐Seytoux 2019). The SAFE coefficient Γ depends on aquifer thickness, hydraulic conductivity anisotropy, riverbed properties (including hydraulic conductivity and entry pressure), and stream geometry. Unlike empirical conductance terms, Γ varies dynamically with stream stage and groundwater head. Full derivations and explanation of the dimensionless conductance are provided by Morel‐Seytoux and co‐workers (Morel‐Seytoux 2009; Morel‐Seytoux et al. 2014, 2017, 2019). The conceptual representation of flow resistance is shown in Figure S1, and the equations and associated flat isotropic conductance parameters used in this study are summarized in Supporting Information Text S1 and Table S2.

Besides the conductance term ($2L\Gamma K_{H}$), another fundamental difference between the SAFE formulation and the FOE formulation (Equation 2) is the definition of the groundwater head threshold at which the stream and aquifer are considered hydraulically disconnected. In the SAFE framework, this threshold is represented by the incipient desaturation head $h_{\text{incip}}$, which reflects the physical conditions under which desaturation begins beneath the streambed and flow becomes independent of further groundwater head decline.

The incipient desaturation head is computed as:

(4) $$h_{\text{incip}}=h_{s}-\frac{W_{\mathrm{per}}}{2K_{H}\Gamma }K_{\mathrm{cl}}\frac{h_{\mathrm{stg}}+e_{\mathrm{cl}}+h_{\mathrm{ce}}}{e_{\mathrm{cl}}}$$

where $h_{\mathrm{stg}}$ is stream stage and $h_{\mathrm{ce}}$ is the entry pressure of the riverbed material. Note that although the parameter $K_{\mathrm{cl}}$ appears in both the FOE and SAFE formulations, its role differs fundamentally between the two approaches. In the FOE formulation, $K_{\mathrm{cl}}$ is typically treated as a lumped constant calibration parameter that represents combined streambed effects, whereas in the SAFE formulation it represents a physical property of the riverbed material, for which representative values are available in the literature (Table S1), although in practice it may still be subject to uncertainty and calibration. More importantly, while $K_{\mathrm{cl}}$ remains constant in both formulations, the effective exchange behavior in SAFE is governed by the resistance term Γ, which varies dynamically with hydrologic conditions, rather than by a single constant conductance parameter as in the FOE approach. The incipient desaturation criterion is consistent with the formation of a groundwater mound beneath the streambed, allowing the stream to remain hydraulically connected even when groundwater heads fall below the riverbed elevation. Under these conditions, the stream may remain hydraulically connected to the aquifer even when groundwater heads fall below the riverbed elevation. Because the formulation of $h_{\text{incip}}$ depends explicitly on the SAFE dimensionless conductance Γ, this disconnection criterion is not transferable to FOE formulations.

#### Asymmetric SAFE Formulation

The SAFE formulation is based on an analytical representation of flow resistance between a stream and the aquifer at a characteristic distance away from the stream, where groundwater flow is assumed to be predominantly horizontal. This concept can be generalized to account for asymmetric groundwater heads on the left and right sides of the stream by allowing the effective flow resistance to differ on each side. Under the SAFE formulation, asymmetry arises from differences in groundwater head at a “far‐enough” distance from the stream, where vertical gradients induced by stream–aquifer interaction diminish and horizontal flow dominates. Miracapillo and Morel‐Seytoux et al. (2014) developed the analytical basis for asymmetric stream–aquifer exchange in finite‐difference models by introducing distinct groundwater heads on either side of the stream (see SI Text S2). In IWFM's finite‐element grid, the mesh discretization naturally places nodes on both left and right sides of the stream, allowing evaluation of distinct groundwater heads on each bank without introducing additional computational elements.

Following Morel‐Seytoux (2009), the “characteristic distance at which groundwater flow becomes effectively horizontal,” denoted $G_{\text{SAFE}}$, is approximated at each stream node as $G_{\text{SAFE}}=2A/\left(L_{F}+L_{R}\right)$, where A is the area of influence associated with the stream node, and $L_{F}$ and $L_{R}$ are half‐distances measured along the stream, between the node and its immediate downstream and upstream neighbors, respectively.

In this implementation, representative groundwater heads on the left ($h_{L}$) and right ($h_{R}$) side of the stream are computed by averaging groundwater head values along a line connecting points located at distance $G_{\text{SAFE}}$ from the stream centerline (Figure S2). These heads are used to calculate distinct dimensionless SAFE conductance coefficients, $\Gamma_{L}$ and $\Gamma_{R}$, for the left and right sides, respectively (see Supporting Information Text S2). The resulting asymmetric stream–aquifer exchange fluxes are expressed as:

(5) $$Q_{S}^{L,R}=LK_{H}\Gamma_{L,R}\left(h_{s}-\max\left(h_{L,R},h_{\text{incip}}\right)\right)$$

where *h*_*s*_ is stream stage, *K*_*H*_ is the horizontal aquifer hydraulic conductivity, and $h_{\text{incip}}$ is the incipient desaturation head defining hydraulic disconnection.

In principle, the total stream–aquifer exchange flux should satisfy:

(6) $${Q_{S}=Q}_{S}^{L}+Q_{S}^{R}$$

However, due to numerical interpolation effects associated with large finite‐element sizes and heterogeneous geometries, small deviations from exact additivity may occur. To ensure strict mass conservation, the asymmetric fluxes are therefore used to compute fractional contributions.

$p^{L}=\frac{Q_{S}^{L}}{\left(Q_{S}^{L}+Q_{S}^{R}\right)},p^{R}=\frac{Q_{S}^{R}}{\left(Q_{S}^{L}+Q_{S}^{R}\right)},$ which are then applied to apportion the total symmetric SAFE exchange flux *Q*_*S*_ as $p^{L}Q_{S}$ and $p^{R}Q_{S}$ on the left and right sides of the stream, respectively. This asymmetric partitioning is performed as a post‐processing step after each simulation time step has fully converged. As a result, the groundwater head solution and overall water balance remain governed by the symmetric SAFE formulation, while the asymmetric decomposition provides additional diagnostic information on the spatial distribution of stream–aquifer exchange across stream banks. It is noted that both higher‐order formulations and FOE‐type methods can represent asymmetric stream–aquifer exchange when the model domain is discretized at sufficient resolution to explicitly resolve surface water bodies in two or three dimensions. In such cases, asymmetry emerges naturally from spatial variability in hydraulic gradients. However, these approaches require substantially refined grids. In contrast, the SAFE asymmetric formulation enables representation of asymmetric exchange even when streams are conceptualized as one‐dimensional features, thereby maintaining computational efficiency and applicability to large‐scale regional models.

#### Numerical Comparison of SAFE and FOE Using a Controlled Numerical and Regional‐Scale Test Case

The symmetric and asymmetric SAFE formulations were applied to a controlled numerical test case designed to examine how alternative parameterizations of stream–aquifer exchange influence simulated groundwater heads and exchange fluxes at the scale of a typical groundwater basin. The numerical implementation of SAFE was independently verified against an analytical benchmark prior to conducting the comparison experiments presented here. The verification model configuration and comparison of the numerical and analytical solutions are presented in Supporting Information Text S3 and Figures S7 and S8. The results confirmed that the SAFE implementation reproduces the expected analytical behavior over a range of stream stages. The stage dependence of the effective SAFE conductance is shown in Figure S9.

**Figure 1:**
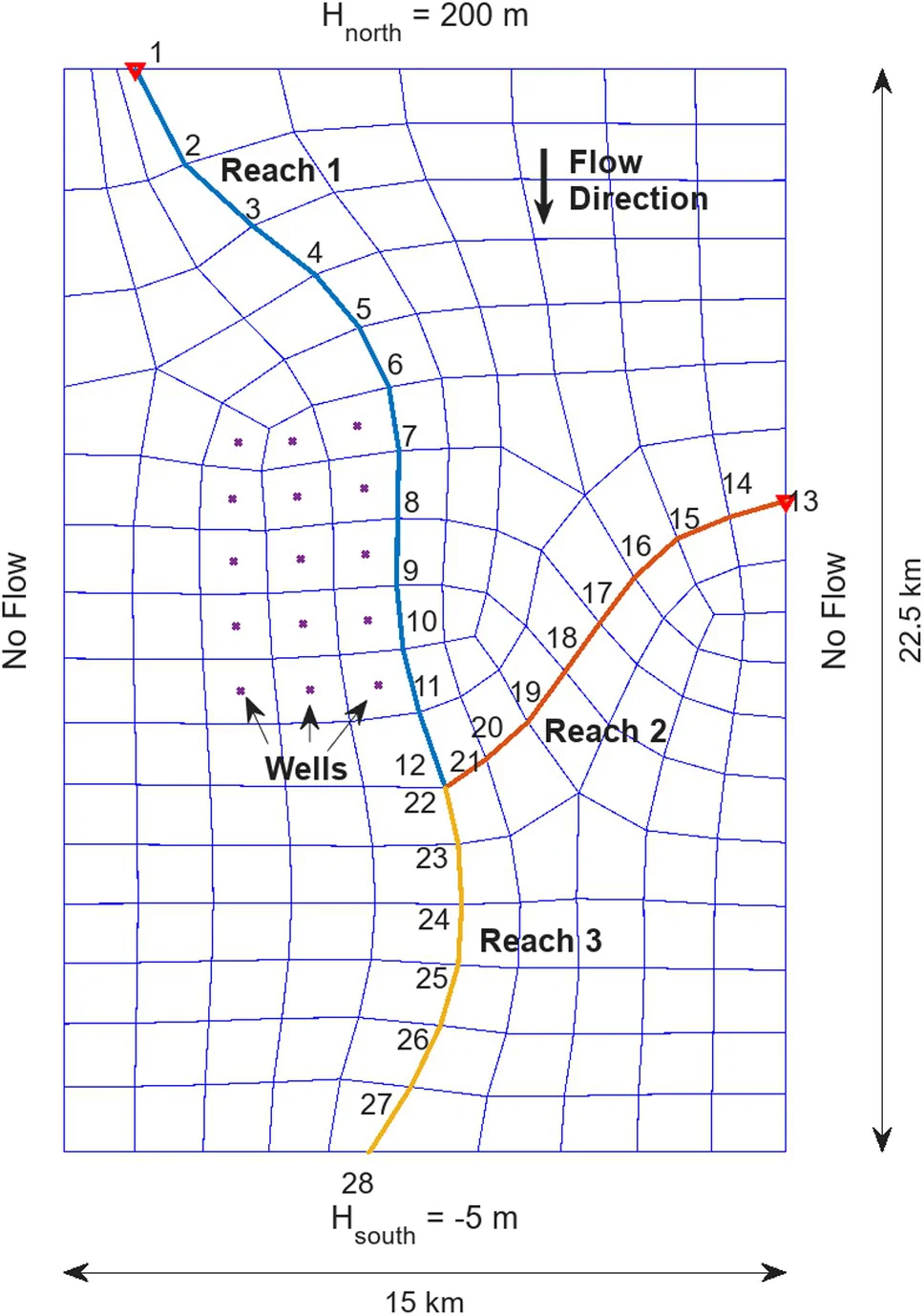
Description of the hypothetical domain, main aquifer features and boundary conditions.

Following this verification, the SAFE and FOE formulations were evaluated using the controlled numerical test case described below. The model domain is rectangular, with dimensions of 15 km by 22.5 km in the x‐ and y‐directions, respectively. The base of the aquifer is set 100 m below ground surface. Constant‐head boundaries of 200 and −5 m are imposed along the northern and southern boundaries, respectively, inducing regional groundwater flow from north to south, while the eastern and western boundaries are treated as no‐flow boundaries. The domain is discretized horizontally into 175 quadrilateral finite elements with an average area of 2 km^2^ and vertically into a single aquifer layer. Three stream reaches are represented within the domain. Reach 1 enters at the northern boundary with an inflow rate of 3.764 m^3^/s and Reach 2 enters from the eastern boundary with an inflow of 1.673 m^3^/s; both discharge into Reach 3. Pumping wells are located on the western side of Reach 1 (Figure 1). Simulations were conducted first without pumping and subsequently with a total combined pumping rate of 1 million m^3^/month for all 15 wells, corresponding to approximately 2190 m^3^/d per well. The horizontal aquifer hydraulic conductivity was assumed uniform at 20 m/d, while streambed hydraulic conductivity and thickness were set to 0.0144 m/d (representative of clay; Table S1) and 1 m, respectively. Simulations to compare the FOE and SAFE formulations were performed within the IWFM framework.

In a second application, the symmetric and asymmetric SAFE formulations are implemented in C2VSimFG, the California Central Valley integrated model, to assess how exchange parameterization influences simulated behavior at the regional scale.

The Central Valley (CV) is an intensively managed agricultural basin covering approximately 52,000 km^2^ in California. Extensive surface water infrastructure—including reservoirs, canals, and regulated river reaches—has substantially altered the natural hydrologic regime, increasing summer flows and reducing winter flows in many rivers (The Nature Conservancy 2014). Combined with a Mediterranean climate and widespread irrigation pumping, these conditions result in predominantly losing stream reaches across much of the valley floor, particularly along tributaries entering from the Coast Range and Sierra Nevada Mountains (The Nature Conservancy 2014).

Several integrated groundwater–surface water models have been developed for the CV, including basin‐wide models (e.g., CVHM—Faunt 2009; CVHM2—Faunt et al. 2024) and regional submodels (Ruud et al. 2004; Phillips et al. 2015; Casillas‐Trasvina et al. 2025). These models employ conductance‐based stream–aquifer interaction formulations analogous to the MODFLOW RIV. The California Central Valley Groundwater–Surface Water Simulation Model, Fine Grid (C2VSimFG), developed by the California Department of Water Resources (DWR), is a finite‐element integrated model used extensively to support groundwater sustainability planning under the Sustainable Groundwater Management Act (SGMA 2014; Miro and Famiglietti 2019; Garner et al. 2020). C2VSimFG is based on IWFM (Dogrul and Kadir 2023) and discretizes the CV into 32,537 finite elements horizontally and four aquifer layers vertically, operating on a monthly time step from 1973 to 2015.

**Figure 2:**
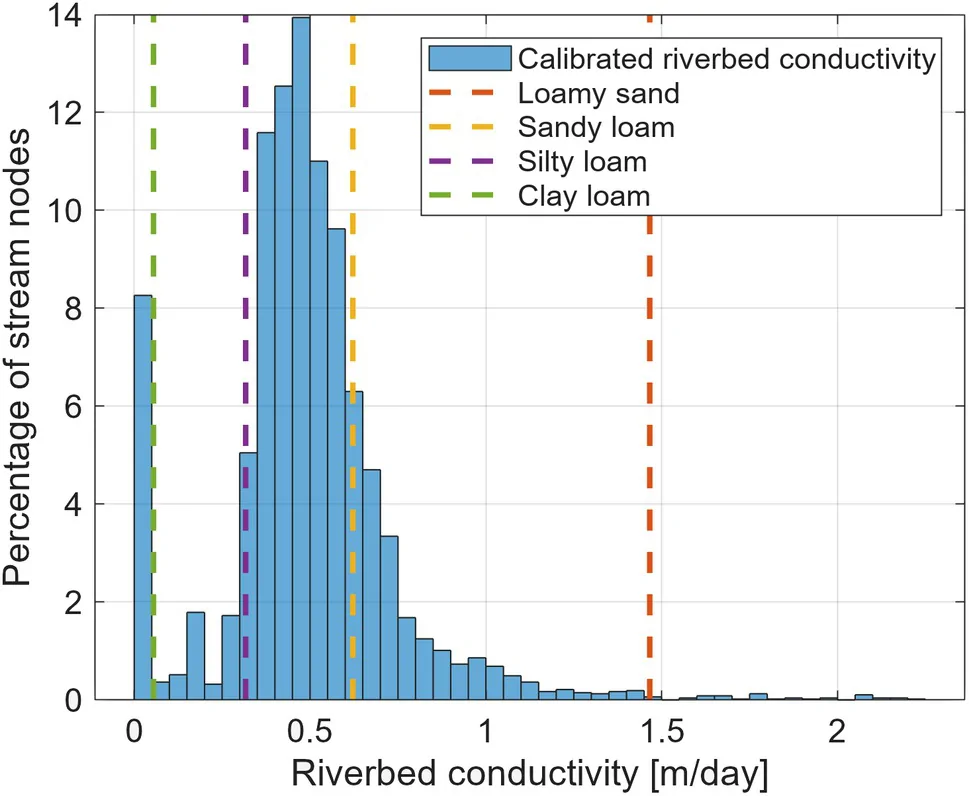
Calibrated riverbed hydraulic conductivity values against the range of streambed material values from Table S1.

In the standard configuration, C2VSimFG represents stream–aquifer exchange using the FOE formulation, assuming a uniform streambed thickness and prescribed wetted perimeter values that remain constant over time. Streambed hydraulic conductivity values were estimated during model calibration (CADWR 2020). As shown in Figure 2, the calibrated conductivities span a wide range corresponding broadly to clay through sandy loam material, with approximately 8% of stream nodes assigned zero conductivity.

For this study, the SAFE formulation was implemented within the existing C2VSimFG framework without recalibration of any parameters. All groundwater, surface water, and stream geometry inputs were kept identical between simulations. Consequently, differences between SAFE and FOE results reflect differences in stream–aquifer exchange parameterization and disconnection criteria, rather than parameter fitting. The SAFE results presented here should therefore be interpreted as uncalibrated numerical outcomes.

## Results

### Comparison of SAFE and FOE in a Controlled Numerical Test Case

**Figure 3:**
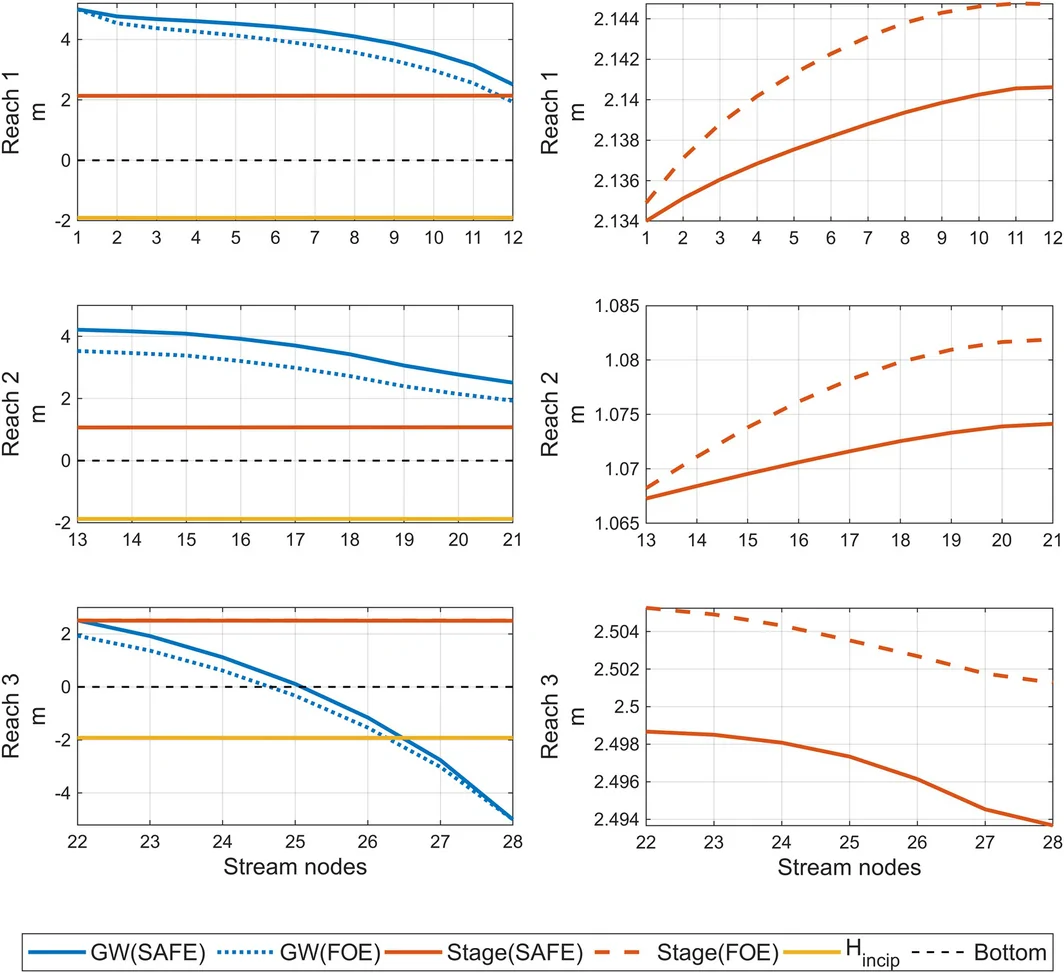
Comparison of groundwater heads (left column) and stream stages (right column) for the three reaches of the hypothetical example comparing the SAFE and FOE formulation without pumping. Plots also show the streambed elevation and the incipient desaturation head used in the SAFE formulation.

The SAFE formulation generally yields higher simulated groundwater heads at stream nodes relative to FOE (Figure 3), with the largest differences (up to ~0.7 m) occurring along Reach 2, which is the least influenced by imposed head boundaries. In the IWFM implementation used here, stream stage is computed from rating tables that relate streamflow to stage. Therefore, differences in simulated exchange fluxes between the SAFE and FOE formulations lead to small differences in streamflow which are reflected in the computed stream stage. Overall, simulated stream stages are slightly higher under the FOE formulation, although these differences are small (0 to 0.007 m) and unlikely to be hydrologically significant at this scale.

Along Reaches 1, 2, and 3 up to node 25, groundwater heads exceed streambed elevation. Under the FOE formulation, at Reach 3 beginning at node 25, this condition triggers stream–aquifer disconnection. In contrast, the SAFE formulation uses the incipient desaturation head as the disconnection criterion, resulting in continued hydraulic connection until node 26.

**Figure 4:**
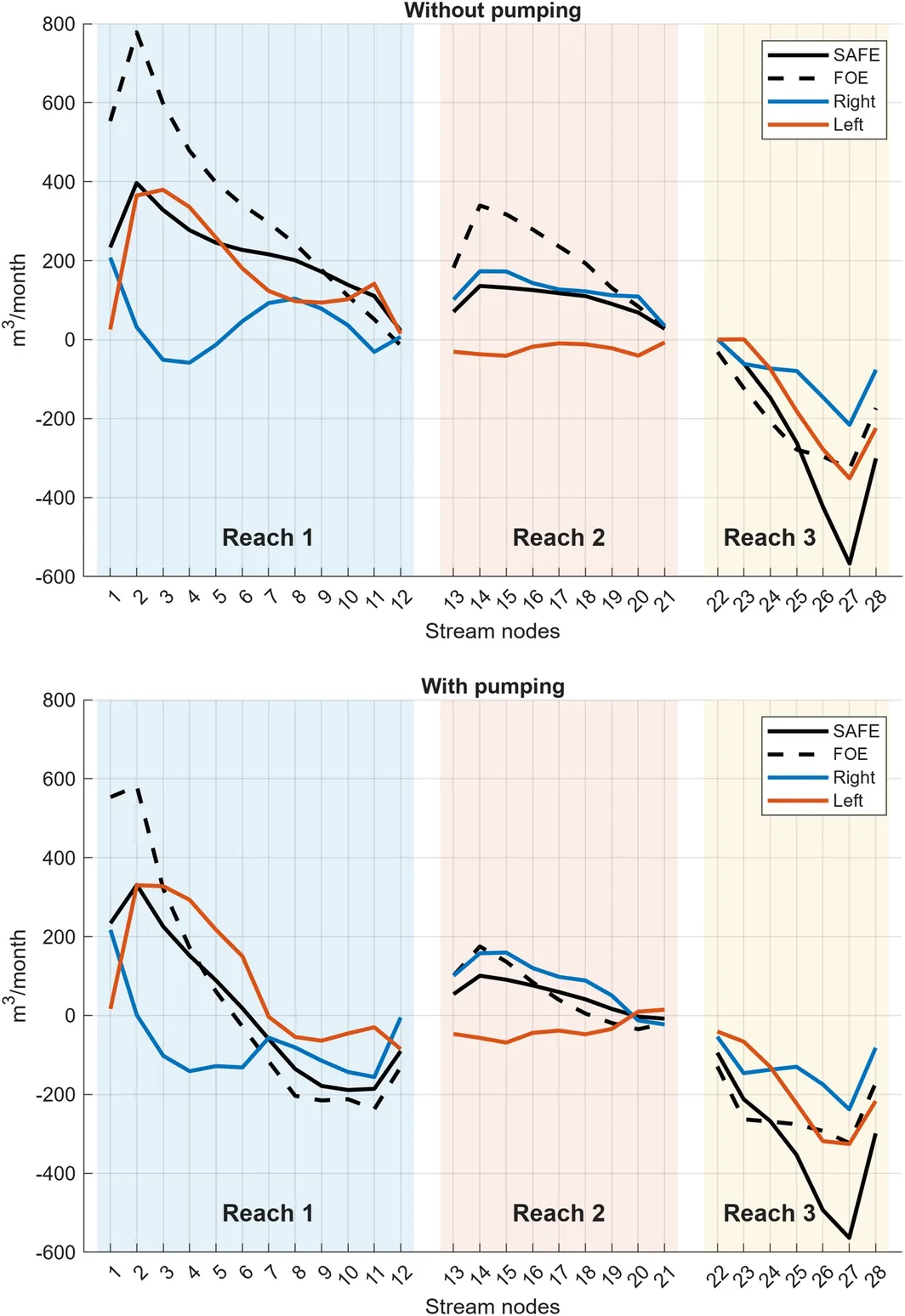
Comparison of stream–groundwater interaction estimated with the SAFE and FOE formulation and the asymmetric head quantification. The black solid and dashed lines correspond to the symmetric SAFE and FOE solutions, while the red and blue lines show the asymmetric stream–groundwater interaction quantification of the left and right side using the SAFE formulation. The top panel corresponds to the simulation without pumping and the bottom panel corresponds to results with pumping enabled. Positive values indicate groundwater return flow (RF) to the stream, while negative values indicate seepage discharge (SPD) from the stream to the aquifer.

There occurred substantial differences in simulated exchange at several stream nodes (Figure 4). For upstream nodes along Reaches 1 and 2 (e.g., nodes 1 to 4 and 13 to 17), the FOE formulation produces nearly twice the RF estimated by SAFE. These differences gradually diminish approaching the confluence of the three reaches and reverse in portions of the losing part of Reach 3, where SAFE produces higher RF than FOE. At nodes where groundwater heads are constrained by boundary conditions and stream‐stage, differences between the two methods are negligible (e.g., nodes 1 and 28), differences in simulated exchange can be attributed primarily to differences in the conductance formulation. Because the wetted perimeter and area of influence are identical in both approaches, these results isolate the role of conductance parameterization in controlling exchange magnitude. Under the FOE formulation, matching SAFE‐derived exchange rates would require adjustment of the empirical conductance parameter, which in practice, may fall within the physically plausible ranges depending on calibration and local conditions. In contrast, the SAFE conductance is derived from aquifer and streambed properties with physical meaning, and varies dynamically with hydrologic conditions, although it may involve additional parameters (e.g. air entry pressure) that carry physical meaning and can in principle be estimated from soil and aquifer properties. At other stream nodes, differences between the two approaches reflect a combined influence of conductance formulation and differences in groundwater and stream heads arising from their distinct connectivity criteria. At a reach scale, the SAFE approach estimates RF volumes that are approximately 35% and 55% lower than those obtained using the FOE method for reaches 1 and 2, respectively. In contrast, for reach 3, the SAFE‐based estimate of seepage discharge is approximately 15% higher than the corresponding FOE estimate (Figure S3).

In the asymmetric SAFE formulation shown in Figure 4, the left–right asymmetry arises primarily from the imposed regional groundwater flow direction. Reach 2 traverses the domain diagonally, resulting in higher groundwater heads on the northern (right) side of the stream relative to the southern (left) side of stream. Consequently, although Reach 2 is net‐gaining overall, the right side contributes more RF to the stream, while the left side exhibits localized losing conditions. Because the total exchange is conserved, the enhanced contribution from the right side offsets losses from the left.

Reach 1 exhibits spatially variable asymmetry due to its meandering geometry. In the upstream segment (nodes 1 to 5), the stream orientation relative to regional flow results in greater contribution from the left side. Downstream, where the reach aligns more closely with the regional flow direction, left‐ and right‐side contributions converge. Reach 3 is predominantly losing but remains hydraulically connected except near its downstream end; the asymmetric formulation indicates that approximately 63% of stream losses are directed toward the left side of the aquifer.

Introducing pumping alters both the magnitude and spatial distribution of stream–aquifer exchange. Pumping on the right side of Reach 1 shifts portions of the reach from gaining to losing conditions. Under pumping, approximately 66% of stream losses are directed toward the right side of the aquifer, reflecting localized drawdown effects. Although pumping is not collocated with Reach 3, regional drawdown influences exchange partitioning along that reach as well, with approximately 58% of stream losses directed toward the left side.

### Comparison of SAFE and FOE for the Central Valley Aquifer System

#### Basin‐Wide Temporal Behavior of Stream–Aquifer Exchange

**Figure 5:**
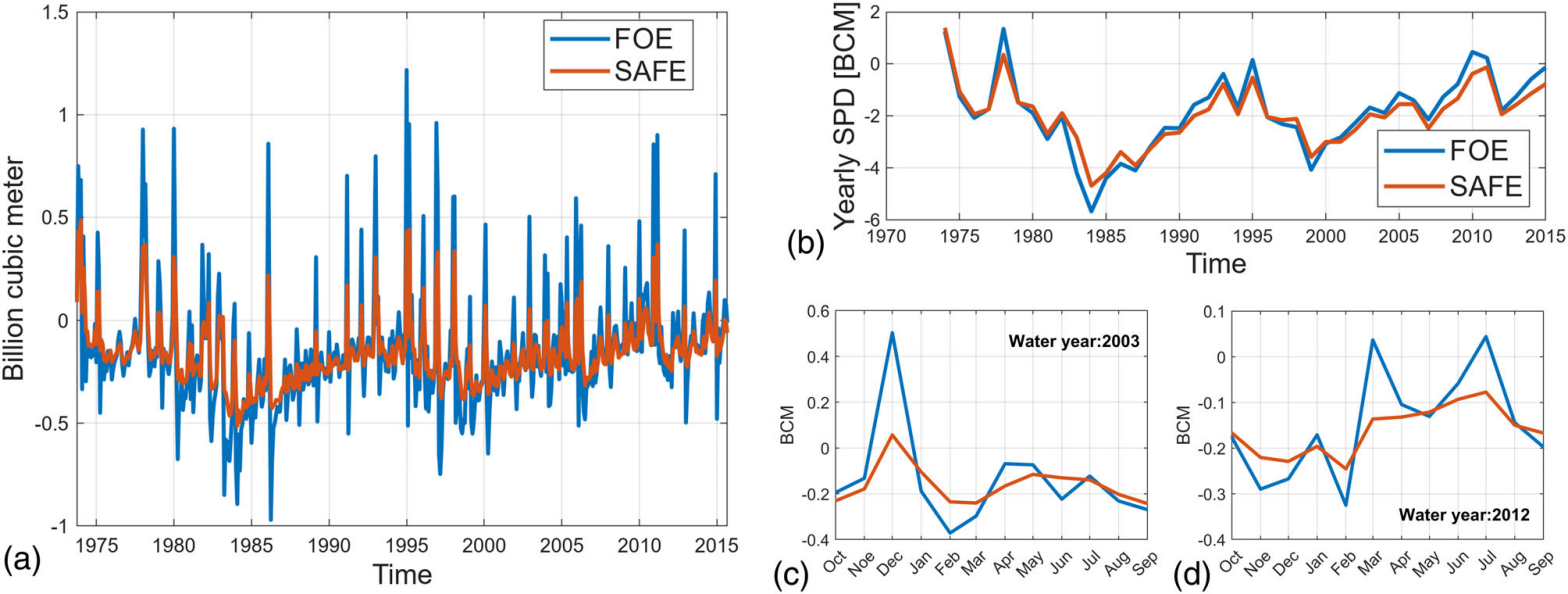
Cumulative analysis of stream–aquifer interaction across all stream nodes. Comparison of monthly flow volumes between the FOE and SAFE formulations (a), annual flow volumes (b), and monthly flow volumes for water years 2003 and 2012 (c,d). Despite differences in temporal variability, both methods yield similar cumulative annual exchange volumes.

We first evaluated basin‐wide stream–aquifer exchange by computing cumulative monthly RF and seepage discharge across all stream nodes for each simulation time step is a methods statement. Replace with: Monthly exchange totals differ substantially in variability between formulations despite similar annual cumulative volumes (Figure 5). The FOE formulation produces extreme oscillations in monthly RF and seepage discharge, with values ranging between +1 and −1 billion cubic meters (BCM) per month. In contrast, SAFE yields a narrower range of exchange volumes that generally remain within ±0.5 BCM. This difference reflects the dynamic conductance behavior in SAFE, which varies continuously with hydraulic conditions, as opposed to the fixed conductance used in FOE.

To illustrate this contrast under different hydrologic conditions, Figure 5c and 5d present monthly exchange volumes for water year 2003 (a normal precipitation year) and 2012 (a dry year). In both years, the FOE formulation exhibits sharp month‐to‐month oscillations, including rapid reversals from strong RF to strong seepage discharge. These oscillations persist even during summer months when hydrologic forcing changes gradually. In contrast, SAFE produces smoother transitions from winter RF to summer seepage discharge, consistent with gradual seasonal changes in heads and streamflow. Despite these differences at the monthly scale, both methods yield very similar cumulative annual exchange volumes (Figure 5b). This agreement indicates that the principal distinction between SAFE and FOE lies in the temporal and spatial distribution of exchange rather than in long‐term mass balance.

#### FOE vs. SAFE: Connectivity Classification and Longitudinal Exchange Variability

Spatial patterns are assessed at the final simulation time step (September 2015) for all stream nodes. Stream nodes are classified into three interaction states: gaining, losing but connected, and losing disconnected, per the SAFE incipient desaturation criterion.

**Figure 6:**
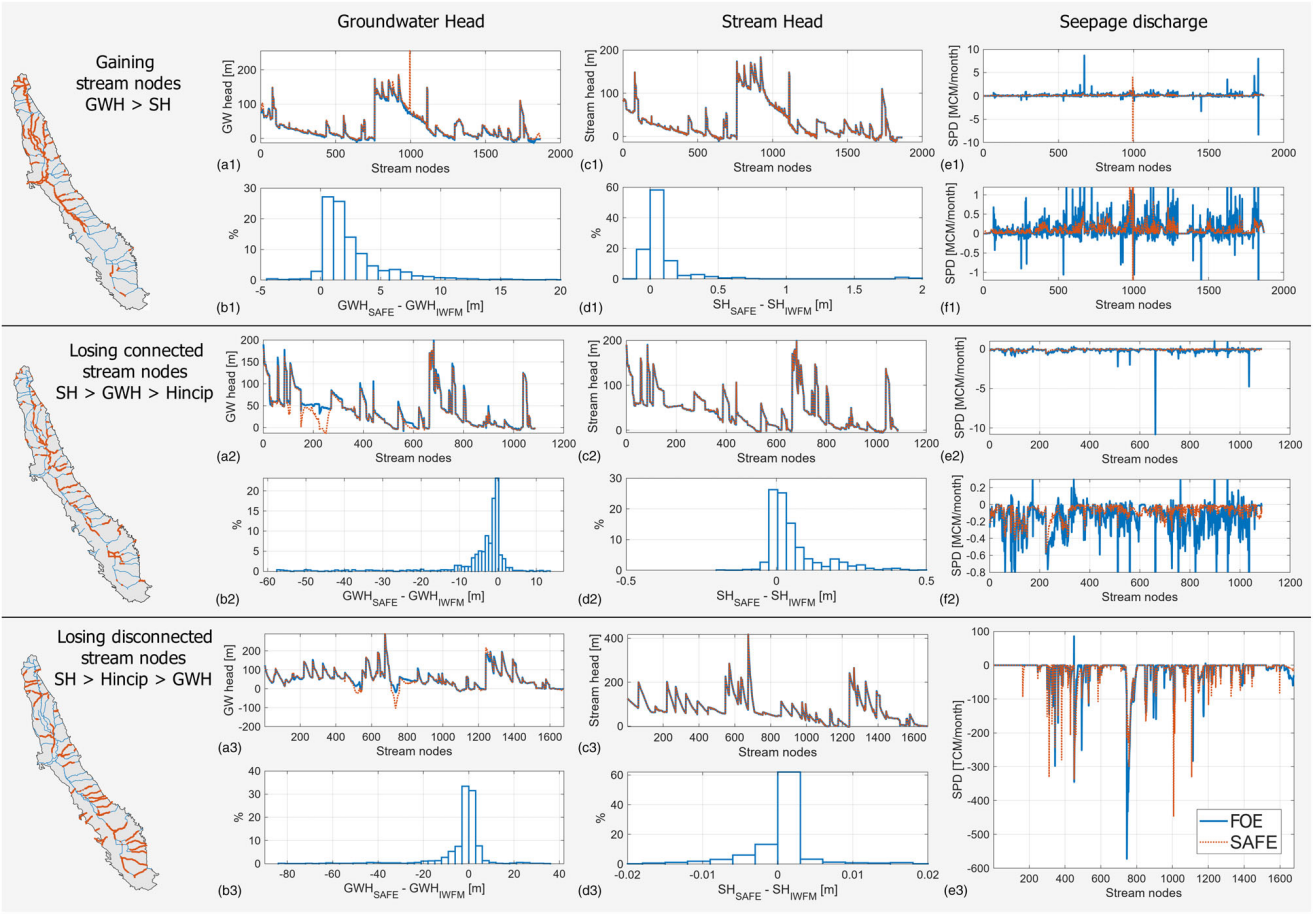
Comparison of the FOE and SAFE formulations for the last step of the C2VSimFG simulation. The comparison is differentiated based on the state of stream–aquifer interaction, which is determined by the relation between the groundwater head (GWH), stream head (SH), and incipient desaturation head (h_incip_): (1) gaining stream nodes (top row), (2) losing connected stream nodes (middle), (3) losing disconnected stream nodes (bottom). For the groundwater (a1‐a3) and stream head plots (c1‐c3), we show the head as a line plot and the differences between the FOE and SAFE formulations as histogram (b1‐b3, d1‐d3). For the seepage discharge we show the full range of values (e1‐e3) (top graph) and a close‐up (f1‐f3) to highlight the difference in the variability between the two methods.

For gaining stream nodes (Figure 6, top row), groundwater heads exceed stream stage and RF from the aquifer to the stream dominates. Using the SAFE formulation, approximately 40% of stream nodes are classified as gaining, compared to 30% under the FOE formulation, with most nodes located in the northern part of the Central Valley. SAFE generally produces higher groundwater heads at these nodes than FOE (Figure 6 a1, b1), with approximately 80% of nodes exhibiting differences of up to 5 m and a small fraction (~3%) exceeding 10 m. These differences can be attributed to the distinct exchange formulations attributable to FOE's fixed linear flux imposing stronger localized gradients than SAFE's physically based resistance. Differences in the simulated stream stage are comparatively minor (Figure 6 c1, d1), with more than 96% of nodes differing by less than 0.5 m. RF magnitudes are broadly similar for most nodes (Figure 6 e1, f1), although a limited number of nodes exhibit substantially larger discrepancies (up to ~8000 m^3^/month). These localized differences arise primarily from differences in the effective conductance formulation rather than from differences in hydraulic gradients, as boundary‐controlled stream and groundwater heads are nearly identical for both methods at many of these locations.

**Figure 7:**
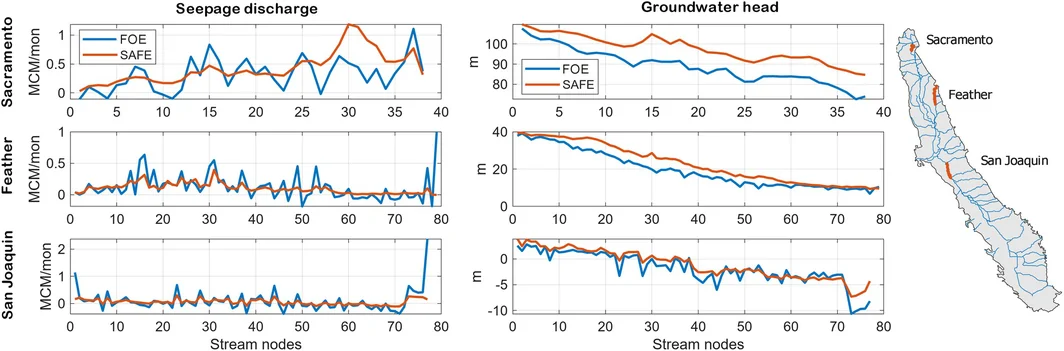
Comparison of the stream–aquifer interaction states and estimated groundwater heads between the FOE and SAFE formulations for three selected CV streams.

Along the San Joaquin River (Figure 7, top row), groundwater heads simulated using the FOE formulation oscillate by up to approximately 3 m between adjacent stream nodes, whereas SAFE produces smoother head profiles with variations typically within 1 m. For the Feather and Sacramento rivers, both formulations simulate comparatively smooth downstream groundwater head gradients with limited node‐to‐node variability.

In contrast to the groundwater head behavior, simulated seepage discharge exhibits substantially greater longitudinal variability under the FOE formulation along all three reaches. For example, stream nodes 14/15 and 49/50 along the Feather River are located only 800 and 600 m apart, respectively, yet the FOE formulation produces differences in seepage discharge of 550 and 630 m^3^ month^−1^ between these adjacent nodes. Such abrupt node‐to‐node variability cannot be readily explained by local hydrogeologic or hydraulic conditions at this spatial scale which exhibits a relatively low variability (Figure S6). The SAFE formulation, by comparison, yields smoother longitudinal variations in seepage discharge between neighboring stream nodes.

Despite these differences in local variability, both methods reproduce similar reach‐scale spatial patterns of stream–aquifer exchange. For both formulations, seepage discharge remains approximately constant along the San Joaquin River, increases progressively downstream along the Sacramento River, and increases up to approximately node 30 before declining along the Feather River. Under the FOE formulation, large seepage discharge at one stream node is frequently followed by substantially lower values at adjacent nodes, indicating compensatory oscillations that preserve reach‐scale water balance; SAFE distributes exchange more evenly, producing the same reach totals with less node‐to‐node variability (Figure 7).

Approximately 23% of CV stream nodes are classified as losing but connected, a state unrepresentable in the FOE formulation, which triggers disconnection at the riverbed elevation rather than at the incipient desaturation head. These nodes are primarily located along the main Sacramento and San Joaquin Rivers, with additional occurrences distributed throughout the basin (Figure 6, middle row). For these nodes, SAFE simulates groundwater heads that are lower than those produced by the FOE formulation at approximately 80% of locations, with differences reaching up to 15 m, while the remaining 20% of nodes exhibit higher heads under SAFE (Figure 6 a2, b2). Notably, for a subset of nodes (approximately nodes 150 to 270 in Figure 6 a2), head differences between the two formulations range from 20 to 60 m. These nodes are classified as connected under SAFE, but disconnected under FOE, leading to fundamentally different stream–aquifer interaction regimes despite identical boundary conditions and forcing. Differences in simulated stream heads between the two formulations remain small for losing but connected nodes, typically within ±0.5 m (Figure 6 c2, d2). However, marked differences are observed in the spatial variability of seepage discharge. The FOE formulation produces pronounced node‐to‐node oscillations in exchange fluxes, ranging from approximately −800 to 200 m^3^/month, whereas SAFE yields a narrower and smoother range of −400 to 0 m^3^/month (Figure 6 e2, f2). These differences arise from the contrasting treatment of conductance and disconnection thresholds rather than from differences in hydraulic forcing.

About 36% of all stream nodes are classified as ‘losing and disconnected’ under the SAFE formulation, predominantly located in the southern CV and along tributaries to the main rivers. For these nodes, groundwater head differences between SAFE and FOE are generally larger than for gaining or losing‐but‐connected nodes. SAFE simulates lower groundwater heads than FOE at approximately 52% of disconnected nodes, with differences up to 20 m, while a smaller fraction (about 5%) exhibits differences of up to 80 m (Figure 6 a3, b3). Conversely, FOE produces higher groundwater heads at approximately 38% of nodes, typically by less than 5 m, with a small subset (approximately 5%) showing differences up to 40 m. Stream heads are nearly identical between the two formulations for disconnected nodes (Figure 6 c3, d3), and both approaches produce comparable seepage discharge magnitudes and longitudinal variability (Figure 6 e3). This convergence is expected, as both formulations rely on the same analytical solution under fully disconnected (unsaturated) conditions.

Overall, the principal distinction between the FOE and SAFE formulations at the regional scale lies in the identification and treatment of transitional connectivity states. By using the incipient desaturation head rather than the riverbed bottom elevation as the disconnection criterion, SAFE classifies a smaller fraction of stream nodes as disconnected (36%) compared to FOE (45%). This difference leads to systematic changes in simulated groundwater heads and to smoother spatial distributions of exchange fluxes, while preserving comparable basin‐scale water balances.

### Demonstrating the Unique Numerical Capability of Asymmetric SAFE Quantification

This section demonstrates the numerical capability of the asymmetric SAFE formulation within a large‐scale integrated groundwater–surface water model. The objective of this exercise is to illustrate how the implemented asymmetric SAFE formulation distributes stream–aquifer exchange between opposing stream banks under complex hydrologic forcing and heterogeneous management conditions.

**Figure 8:**
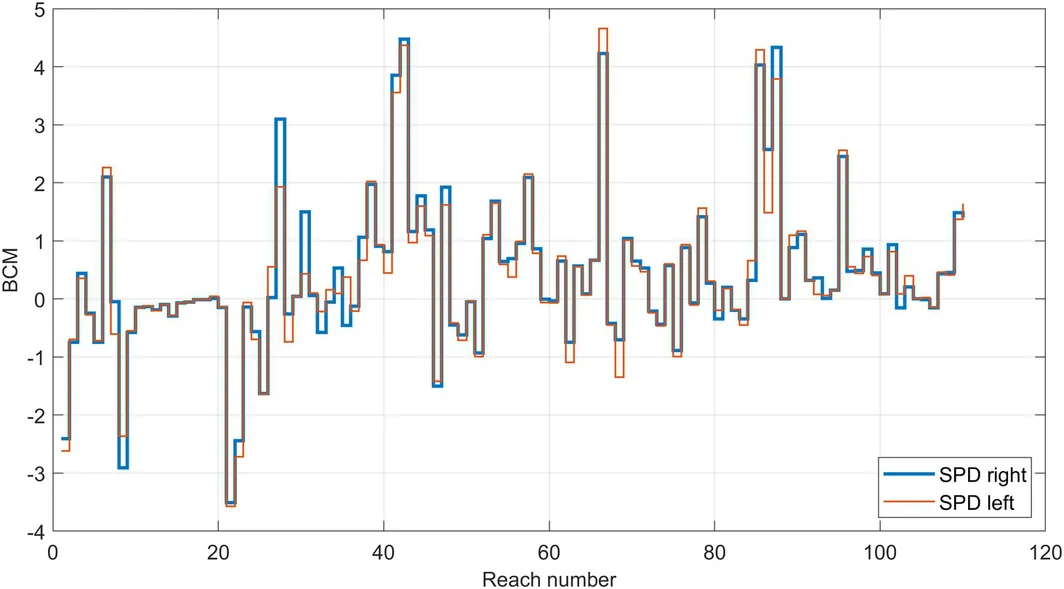
Total seepage discharge on the left and right sides of each stream reach estimated for 1975 to 2015 using the asymmetric SAFE formulation. Values represent cumulative volumes aggregated by reach.

The IWFM C2VSimFG stream network consists of 110 stream reaches. Figure 8 summarizes the cumulative left‐ and right‐bank seepage discharge for each reach over the 1975 to 2015 simulation period. For most reaches (approximately 95%), cumulative left–right differences remain between −0.5 and 0.5 BCM (−11,900 to 11,900 m^3^/year), indicating that, when aggregated over entire stream reaches and long simulation periods, asymmetric effects may not be significant at this level of spatial and temporal integration. This behavior does not imply a characteristic spatial threshold at which asymmetry diminishes but rather reflects the averaging of opposing exchange patterns over large spatial extents and long‐time scales. At smaller spatial scales, however, asymmetric exchange can be significant (Figure S4), particularly in settings with strong lateral hydraulic gradients or in proximity to pumping wells. In such cases, representing asymmetric flow may improve characterization of localized stream–aquifer interactions and associated groundwater responses. However, a small number of reaches exhibit substantially larger cumulative differences, reaching up to 1.2 BCM over the 1975 to 2015 period. A pronounced example is stream reach 8 (Kings River), where the cumulative left–right difference over the 42‐year simulation period is approximately 1.7 BCM (~40,500 m^3^/year). This reach coincides with administrative boundaries between Groundwater Sustainability Agencies (GSAs) under SGMA, as well as boundaries defined in Bulletin 118. The Kings River flows predominantly from east to west, and the simulated asymmetry indicates that the southern side of the river (left SPD) receives a greater portion of stream recharge over the long term. It should be noted that these differences are spatially localized rather than uniformly distributed along the reach.

**Figure 9:**
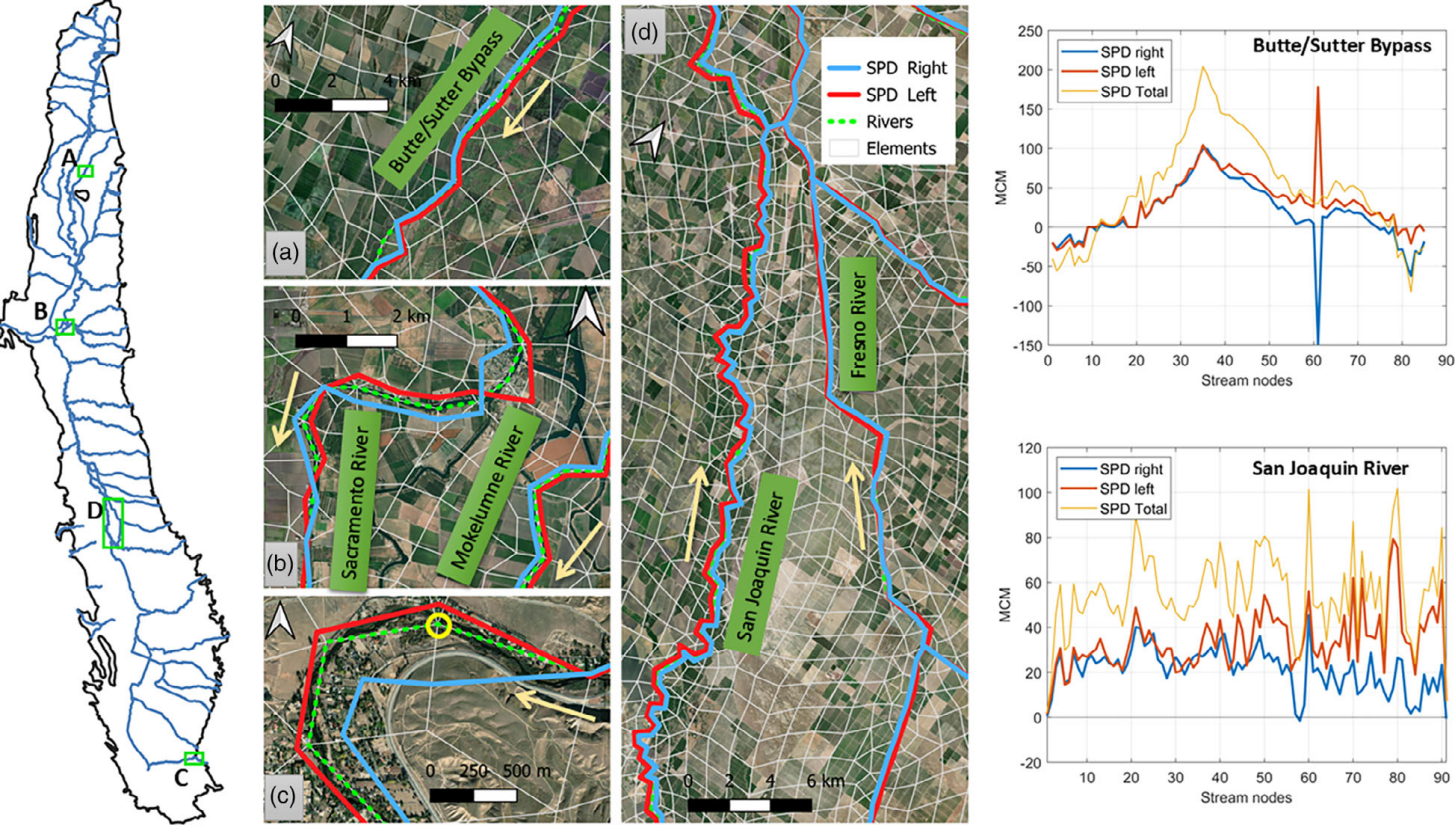
Schematic representation of cumulative asymmetric seepage discharge for selected stream segments. Lateral offsets represent cumulative exchange volume. Right panels show left‐, right‐, and total exchange along selected reaches. Positive offsets denote groundwater return flow to the stream, whereas negative offsets indicate seepage discharge from the stream to the aquifer. Red and blue curves represent left‐ and right‐bank contributions, respectively, with offset magnitude proportional to cumulative exchange volume.

For the stream node highlighted in Figure 9c, the asymmetric SAFE formulation estimates a total cumulative seepage discharge of 92.87 MCM over the simulation period, partitioned into 23 MCM on the left bank and 69.87 MCM on the right bank. This partitioning reflects persistent differences in groundwater heads at the characteristic SAFE distance from the stream. Along the upstream segment of the Kern River (Figure 9c), the first node lies at the model boundary and therefore lacks a right‐bank contribution. Downstream nodes indicate that approximately 75% of total seepage discharge occurs on the right side of the channel.

Figure 9a illustrates a segment of the Butte/Sutter Bypass (Reach 86), one of the reaches exhibiting the left–right differences in Figure 8. While exchange is nearly symmetric in the upstream half of this reach, the downstream half shows a persistent dominance of left‐bank RF, contributing approximately 64% of the total RF. At one location (node 61), left‐bank RF exceeds total RF, requiring compensatory discharge on the right bank to maintain mass balance. Such cases are uncommon but may occur when strong head asymmetry develops across the stream corridor.

Figure 9b shows segments of the Sacramento River (Reach 103) near the Delta and the Mokelumne River (Reach 50). For both reaches, cumulative left‐ and right‐bank contributions are approximately balanced at the reach scale (Figure 8), although local transitions between gaining and losing conditions occur on both banks. Figure 9d presents extended segments of the San Joaquin (Reach 26) and Fresno Rivers (Reach 27). For this system, cumulative seepage discharge on the right bank exceeds the left bank by approximately 1 BCM over the simulation period. While this difference is not visually apparent at map scale, node‐level analysis shows that asymmetry emerges downstream of node 40, where left‐bank contributions exceed right‐bank contributions by 15 to 50 MCM. The observed asymmetry is primarily driven by spatial variability in hydrologic forcing. For example, analysis of average deep percolation over the last 15 years of the simulation (Figure S5) (2000 to 2015) indicates that upstream areas on the right bank receive substantially higher recharge (approximately 200 to 600 mm/year), whereas downstream recharge decreases to approximately 50 mm/year. This contrast generates lateral hydraulic gradients that contribute to asymmetric stream‐aquifer exchange. In addition, spatial variability in groundwater pumping further influences the distribution of exchange fluxes along the reach.

**Figure 10:**
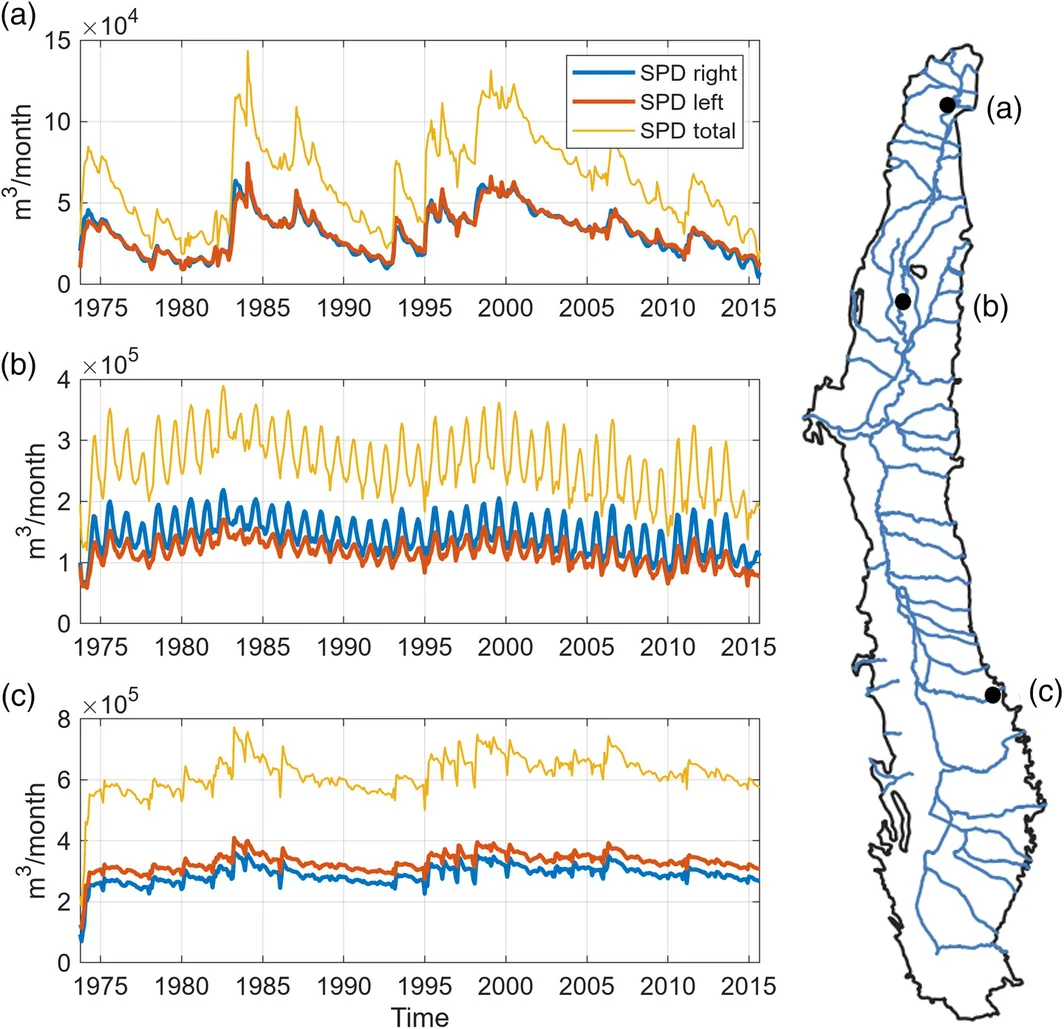
Representative examples of asymmetric seepage discharge time series at individual stream nodes. Node locations (a‐c, ordered north to south) are shown on the inset map.

To further illustrate temporal behavior, Figure 10 shows left‐ and right‐bank exchange time series for three representative stream nodes. Figure 10a presents a node from the Sacramento River where left‐bank contributions dominate while both sides exhibit similar temporal patterns. Figure 10b illustrates a node of Sacramento River where the right bank consistently contributes a larger fraction of exchange and exhibits greater temporal variability. Figure 10c shows a node of San Joaquin River with nearly symmetric partitioning throughout the simulation. These examples demonstrate that asymmetric exchange may arise either from persistent head offsets or from differences in temporal response between opposing banks.

At the regional scale, the asymmetric SAFE results indicate that left–right partitioning of stream–aquifer exchange is primarily controlled by stream orientation relative to regional groundwater gradients, as well as localized stresses such as pumping, irrigation, and managed recharge. While cumulative asymmetric effects are small when aggregated over the entire Central Valley, that is, over stream reaches spanning tens to hundreds of kilometers and over multi‐decadal periods, localized differences can be substantial at individual stream nodes and reaches, typically on the order of hundreds of meters to a few kilometers.

The SAFE formulation approximately doubled overall runtime in the C2VSimFG application, owing to additional iterations required per time step.

## Discussion

In this study, we presented a numerical investigation of the physically based SAFE formulation including its extension for asymmetric flow quantification, implemented within the finite‐element Integrated Water Flow Model (IWFM) framework. The results demonstrate how SAFE's physically derived, state‐dependent conductance alters simulated exchange behavior relative to the empirical FOE approach across both hypothetical and regional‐scale applications.

### Comparison of Physically Based (SAFE) and Empirical (FOE) Stream Conductance Formulations

Systematic differences in simulated exchange between SAFE and FOE arise fundamentally from contrasting representations of streambed conductance. The FOE formulation relies on an empirical leakance factor that aggregates streambed properties and geometric effects into a single parameter that is typically assumed constant in time and often estimated through calibration. The SAFE formulation does not eliminate the need for calibration but rather uses calibration parameters with physical meaning that can be constrained within plausible ranges based on independent information. The temporal oscillations observed in FOE simulations arise from the fixed conductance assumption: as hydraulic conditions shift seasonally, the model compensates by alternating exchange direction between neighboring nodes rather than adjusting the conductance coefficient itself, producing node‐to‐node reversals that are inconsistent with gradually changing boundary conditions. The SAFE formulation avoids this artifact by continuously re‐deriving conductance from evolving head conditions, yielding spatially coherent exchange patterns that are physically consistent with the underlying hydrologic forcing. This distinction is most consequential for applications requiring reliable sub‐annual exchange estimates, for example, monthly groundwater recharge accounting under California's Sustainable Groundwater Management Act (SGMA) or operational conjunctive use planning, where oscillating FOE signals could be misinterpreted as real hydrologic variability rather than numerical artifacts.

### Significance of the Incipient Desaturation Criterion

Unlike the FOE riverbed‐elevation threshold, the incipient desaturation criterion maintains hydraulic connection under conditions where FOE would classify nodes as disconnected, with practical consequences for exchange accounting.

Under FOE, nodes classified as disconnected contribute a seepage rate independent of groundwater head, effectively decoupling exchange from drawdown‐driven head changes. Under SAFE, those same nodes remain head‐dependent because hydraulic connection is maintained through the forming unsaturated zone until the incipient desaturation threshold is reached. In over‐drafted basins where pumping routinely draws heads below streambed elevation, a common condition in the Central Valley, this distinction affects estimates of natural and managed recharge and has direct implications for attributing exchange between adjacent SGMA subbasins whose regulatory boundaries coincide with river corridors.

### Novelty and Implications of Asymmetric Quantification

The asymmetric SAFE implementation partitions exchange between left and right banks based on spatially variable heads, a capability absent from standard packages, with direct implications for management‐unit scale accounting.

The numerical experiments demonstrate that asymmetric exchange patterns are primarily controlled by the orientation of stream reaches relative to groundwater flow gradients and by localized stresses such as pumping, irrigation, or managed recharge. The asymmetric SAFE formulation is therefore broadly applicable, with the greatest value in settings where lateral hydraulic gradients are important, such as near pumping wells, along reaches influenced by uneven recharge, or where streams coincide with management boundaries. Where river corridors coincide with regulatory boundaries, as is common under California's SGMA subbasin delineation framework, accurately partitioning exchange between left and right banks directly affects the estimated water balance of adjacent subbasins and GSA compliance accounting, a gap the asymmetric SAFE formulation fills within a regional‐scale model wherever streams coincide with regulatory boundaries.

### Scope, Limitations, and Future Work

This study was intentionally framed as a numerical implementation and comparative behavior analysis rather than a validation exercise. Accordingly, and consistent with the intended scope no recalibration was performed for the SAFE simulations, and no direct comparison to observed streamflow, groundwater heads, or independent exchange estimates was undertaken. These findings should be interpreted as differences in numerical behavior and sensitivity to parameterization choices, rather than as evidence of improved predictive accuracy. As with any physically based formulation, the reliability of SAFE depends on appropriate parameter selection and where necessary, calibration within physically bounds.

The increased computational complexity of the SAFE formulation resulted in longer simulation runtimes relative to FOE, approximately doubling the total runtime in the Central Valley application. Future work should focus on targeted validation using field observations or independent exchange estimates at smaller scales, as well as exploring strategies to improve computational efficiency. Such efforts would allow a more rigorous assessment of predictive performance of the proposed framework.

Overall, this work establishes a foundation for applying physically based stream–aquifer exchange formulations in large‐scale integrated models and provides a framework for future studies aimed at evaluating their predictive performance under real‐world conditions.

## Conclusions

Exchange formulation choice exerts a first‐order control on the spatial and temporal distribution of simulated stream–aquifer exchange even when long‐term cumulative volumes remain similar. The FOE formulation's fixed empirical conductance used in many groundwater models produces pronounced node‐to‐node oscillations and month‐to‐month reversals in exchange direction, reflecting numerical artifacts of a static parameter under changing hydraulic conditions rather than real hydrologic signals. The SAFE formulation implemented here avoids these artifacts by continuously re‐deriving conductance from evolving head gradients, yielding spatially coherent and temporally smooth exchange patterns consistent with gradual seasonal forcing. This distinction is most consequential for applications requiring reliable sub‐annual exchange estimates, including monthly recharge accounting under SGMA or operational conjunctive use planning.

The incipient desaturation criterion introduces a practically significant third connectivity state: approximately 23% of Central Valley stream nodes are classified as losing but connected under SAFE, nodes that FOE treats as disconnected and therefore head‐independent. Because those nodes remain head‐dependent under SAFE, the distinction directly affects estimates of natural and managed recharge in over‐drafted basins and has implications for attributing exchange across SGMA subbasin boundaries that coincide with river corridors.

The asymmetric SAFE implementation demonstrates that left–right exchange partitioning is primarily controlled by reach orientation relative to regional groundwater gradients and by localized stresses. While cumulative left–right differences are small for most Central Valley reaches over multi‐decadal periods, selected reaches, particularly those coinciding with GSA administrative boundaries, exhibit asymmetries exceeding 1.7 BCM, with direct implications for regulatory water balance accounting.

These results characterize numerical behavior rather than predictive performance: no recalibration was performed and no validation against field observations was undertaken. The implementation framework established here provides a foundation for targeted reach‐scale and field‐scale validation, calibration strategy development, and systematic assessment of when the additional computational cost of SAFE, approximately double that of FOE in the Central Valley application, is warranted relative to simpler conductance‐based approaches.

### Artificial Intelligence (AI) Statement

The authors confirm that no artificial intelligence (AI) tools or generative AI technologies were used in the conception, analysis, interpretation, writing, or preparation of this manuscript.

## Supporting information


**Data S1.** Supporting Information.
**Figure S1**. Illustration of flow resistance between a stream cross section and an aquifer cross section at the “far enough” distance where the flow is horizontal.
**Figure S2**. Estimation of “far‐enough” distance in the finite element implementation. The blue lines are the edges of the finite elements and the red lines represent streams. The orange lines show the $G_{\text{SAFE}}$ distance for the two nodes of the stream segment. The hydraulic head is interpolated along the line that connects the two points at $G_{\text{SAFE}}$ distance from the stream nodes (black dots).
**Figure S3**. Cumulative stream aquifer volume aggregated by reach.
**Figure S4**. Kernel density estimates (KDEs) of the absolute differences between left and right bank stream aquifer exchange across all stream nodes and time steps. Each curve represents the distribution of difference (in thousands cubic meter per month) for a single time step. The results indicate that over the simulation period most stream nodes exhibit differences in exchange magnitude on the order of 10^**2**^ to 10^5^ m^3^/month corresponding approximately to 500 to 100,000 cubic meter per month.
**Figure S5**. Spatial distribution of average deep percolation for the period 2000 to 2015 in the San Joaquin River region. Upstream areas on along the right bank receive substantially higher recharge (approximately 200 to 600 mm/year), whereas downstream recharge decreases to approximately 50 mm/year.
**Figure S6**. Streambed conductance and hydraulic conductivity profiles along three selected rivers. The corresponding stream aquifer exchange behavior is discussed in the main text and presented in Figure 7
**Figure S7**. Numerical grid used for the verification example. Because IWFM does not provide a one‐dimensional simulation option, the analytical 1D problem was represented using a two‐dimensional model domain measuring 1000 m × 500 m and discretized into 10 columns and 5 rows of 100 m × 100 m elements. A stream is located along the left boundary and a constant‐head boundary is assigned along the right boundary. Stream inflow is introduced near the upper‐left corner of the model domain (*y* = 500 m). To minimize the local influence of the inflow point on the comparison with the one‐dimensional analytical solution, groundwater heads from the row farthest from the inflow location (*y* = 0 m) were used for the head‐profile comparison.
**Figure S8**. Comparison of analytical and numerical groundwater head profiles for a low‐stage case ($h_{s}\approx$ 0.3 m; blue) and a high‐stage case ($h_{s}\approx$ 2.0 m; orange). Dots represent numerical SAFE results, dashed lines represent the analytical solution using the assigned streambed conductance (C = 0.0144 m/d), and solid lines represent the analytical solution using the effective conductance calculated internally by SAFE. For the low‐stage case, the effective SAFE conductance is nearly identical to the assigned conductance, resulting in excellent agreement between the analytical and numerical solutions. For the high‐stage case, SAFE computes a substantially larger effective conductance, and agreement is achieved only when this effective conductance is used in the analytical solution.
**Figure S9**. Effective stream–aquifer conductance calculated by SAFE as a function of stream stage. The dashed horizontal line indicates the assigned streambed conductance (0.0144 m/d). At low stream stages, the effective SAFE conductance is very close to the assigned streambed conductance. As stream stage increases, SAFE predicts progressively larger conductance values due to the dependence of stream–aquifer connectivity on wetted perimeter, penetration depth, aquifer geometry, anisotropy, and streambed clogging effects.
**Table S1**. Parameters of riverbed materials. h_ce_ is the entry pressure of the aquifer material and K is the hydraulic conductivity.
**Table S2**. Flat isotropic conductance parameters.

## Data Availability

The Data that support the findings of this study are available in the Hydroshare.org repository (Kourakos et al. 2024).
