# Supplementary material for: Finite Element Implementation of SAFE for Asymmetric Stream‐Aquifer Flow Exchange
Source: Ground Water. 2026 Aug 15;64(5):650–68. doi: 10.1111/gwat.70098 (PMC13571170; doi:10.1111/gwat.70098)
Supplement: Supplementary file 1 [file GWAT-64-650-s001.docx]

**Supporting Information**

**Finite Element Implementation of SAFE for Asymmetric Stream-Aquifer Flow Exchange**

Georgios Kourakos^1^, Hubert Morel-Seytoux^2^, Emin C. Dogrul^3^, Tariq Kadir^3^ Helen E. Dahlke^1^

1 Department of Land, Air and Water Resources, University of California, Davis, 1 Shields Ave, Davis, CA, 95616, USA

2 Hydroprose Consulting International, Santa Rosa, CA 95409, USA

3 California Department of Water Resources, 1516 9th Street, Sacramento, CA 9423695814, USA

**Contents of this file**

Text S1 to S3

Figures S1 to S9

Tables S1 to S2

**Introduction**

This document provides details on the implementation of the SAFE method into the Integrated Water Flow Model (IWFM) (Dogrul and Kadir 2023).

Text S1.

The core of the SAFE method is the SAFE dimensionless coefficient $\Gamma$ which represents the resistance of flow between the stream cross section and the aquifer cross section at some distance “far enough” from the stream channel so that the flow can be assumed horizontal. The resistance of flow is not uniform. We can see (Figure S1) that the water that leaves from the bottom center of the stream cross section have higher flow resistance compared to the ones that leave the stream from the side of the cross section. The SAFE dimensionless coefficient $\Gamma$ is an analytical calculation of the integral of the flow resistance. The derivation and proof of the equation can be found in the works of Morel-Seytoux (2009; 2014; 2018). For completeness of this paper, we provide the equations to calculate the SAFE coefficient $\Gamma$.

Each time step, the groundwater head $H_{gw}$ and the stream head $H_{stream}$ are known from the previous iteration or the initial conditions. The river stage is calculated as $H_{stg}=H_{stream}-{ELEV}_{topRB}$, where ${ELEV}_{topRB}$ is the elevation of the top of the riverbed. In IWFM the wetted perimeter $W_{per}$ is a function of stream stage via rating tables, which are user defined numerical functions between $H_{stg}$ and $W_{per}$ for each stream node. Next the saturated aquifer thickness $D_{aq}$ is calculated as:

$$D_{aq}=H_{gw}-{ELEV}_{bot}+h_{ce}$$

(1)

where ${ELEV}_{bot}$ is the bottom elevation of the first geologic layer. However, we have found that the method is insensitive to the selected groundwater layer therefore in the C2VSimFG application we selected ${ELEV}_{bot}$ as the bottom of the aquifer. $h_{ce}$ is a user-specified value of the entry pressure of water which depends on the riverbed material (Table S1).

Next, SAFE calculates a normalized saturated aquifer thickness $d_{p}^{N}$ and normalized wetted perimeter $W_{p}^{N}$:

$$d_{p}^{N}=\frac{H_{stg}}{D_{aq}}$$

(2)

$$W_{p}^{N}= \frac{W_{p}}{D_{aq}}$$

(3)

Next, the flat isotropic conductance is calculated as follows:

$$\kappa=e^{-\pi\frac{W_{p}^{N}}{2}}$$

(4)

$$\Gamma_{flat}=\frac{1}{2\left( 1+\frac{1}{\pi}\ln\left( \frac{2}{1-\kappa} \right) \right)}$$

(5)

$$\Gamma_{iso}=\Gamma_{flat}\left( 1+a_{1}d_{p}^{N}+a_{2}{d_{p}^{N}}^{2} \right)$$

(6)

where $\alpha_{1}$and $\alpha_{2}$ are coefficients that depend on the normalized saturated aquifer thickness and the normalized wetted perimeter, respectively (Table S2). The SAFE coefficient $\Gamma_{iso}$ is valid when (1) the grid quarter size corresponds exactly to the location of the standard anisotropic far distance, (2) the aquifer is isotropic, and (3) there is no riverbed clogging layer.

In the subsequent calculations the $\Gamma_{iso}$ value is adjusted to generalize the SAFE implementation without the need for the previous assumptions.

For isotropic aquifers the correction for the grid size $\Gamma_{iso\_\Delta}$ is calculated as follows:

$$\Gamma_{iso\_\Delta}=\frac{\Gamma_{iso}}{1+\Gamma_{iso}\frac{\Delta}{D_{aq}}}$$

(7)

where $\Delta=0.25G_{safe}-B_{safe}-{2D}_{aq}$.	The parameter $G_{SAFE}$ depends on the mesh element geometry $G_{SAFE}=2A/\left( L_{B}+L_{F} \right)$ where $A$ is the area of influence of the stream node and $L_{B},L_{F}$ are the stream segments half lengths before and after the stream node respectively. The parameter $B_{SAFE}$ is half the river width $W_{riv}$, $B_{SAFE}=0.5W_{riv}$. The river width in general depends on the geometry of the river cross section. In the IWFM implementation we calculate an equivalent river width under the assumption of a rectangular cross section with $W_{riv}$ = $W_{p}$ - 2$H_{stg}$.

For anisotropic aquifers the equations involve the anisotropy factor $\varrho_{anis}$:

$$\varrho_{anis}=\sqrt{\frac{K_{v}}{K_{h}}}$$

(8)

$$\Delta_{anis}=2\left( \frac{1}{\varrho_{anis}}-1 \right)$$

(9)

$$\xi=\left( 1-\sqrt{D_{p}^{N}} \right)\left( 1-\varrho_{anis} \right)$$

(10)

$$\rho_{f}=1-0.333\xi-0.294\xi^{2}$$

(11)

where $K_{h}$ and $K_{v}$ are the aquifer horizontal and vertical hydraulic conductivity at the stream node location. Finally, the correction for the anisotropy is calculated as:

$$\Gamma_{iso_{\Delta_{anis}}}=\frac{\Gamma_{iso}}{1+\Gamma_{iso}\Delta_{anis}}$$

(12)

$$\Gamma_{anis}={\rho_{f}\Gamma}_{iso_{\Delta_{anis}}}$$

(13)

$$\Delta=0.25G_{safe}-B_{safe}-\frac{{2D}_{aq}}{\varrho_{anis}}$$

(14)

$$\Gamma_{anis-\Delta_{G}}=\frac{\Gamma_{anis}}{1+\Gamma_{anis}\frac{\Delta}{D_{aq}}}$$

(15)

The last correction that accounts for the presence of the riverbed clogging layer is applied on $\Gamma_{iso\_\Delta}$ for isotropic aquifers or on $\Gamma_{anis-\Delta_{G}}$ for anisotropic aquifers.

$$\Gamma_{Q}=\frac{\Gamma_{iso\_\Delta}}{1+ \Gamma_{iso\_\Delta}\frac{K_{h}}{K_{cl}}\frac{e_{cl}}{B_{safe}+H_{stg}}}$$

(16)

where $K_{cl}$ is the hydraulic conductivity of the riverbed material. For anisotropic aquifers we use the $\Gamma_{anis-\Delta_{G}}$ value instead of $\Gamma_{iso\_\Delta}$.

Text S2

For the asymmetric calculation equations 1-5 are similar to the symmetric case. Then using the interpolated head, we differentiate between the left and right flat conductance as:

$${\Gamma_{flat}}_{L}=\left( \frac{1}{H_{stream}-h_{L}} \right)\left\{ \Gamma_{flat}\left( H_{stream}-H_{gw} \right)+ \frac{\left( h_{R}-h_{L} \right)D_{aq}}{4D_{aq}+2B_{safe}} \right\}$$

(17)

$${\Gamma_{flat}}_{R}=\left( \frac{1}{H_{stream}-h_{R}} \right)\left\{ \Gamma_{flat}\left( H_{stream}-H_{gw} \right)- \frac{\left( h_{R}-h_{L} \right)D_{aq}}{4D_{aq}+2B_{safe}} \right\}$$

(18)

Next using the left ${\Gamma_{flat}}_{L}$ and right ${\Gamma_{flat}}_{R}$ we repeat the calculations of eq. 6 – 15 twice to calculate the left and SAFE dimensionless coefficient without the correction for the clogging layer.

For isotropic aquifers the clogging layer correction is expressed in a similar manner to Eq. 16:

$${\Gamma_{Q}}_{L}={\Gamma_{iso\_\Delta}}_{L}\left( 1+{\Gamma_{iso\_\Delta}}_{L}\frac{K_{h}}{K_{cl}}\left( \frac{e_{cl}}{B_{safe}+H_{stg}} \right) \right)$$

(19)

$${\Gamma_{Q}}_{R}={\Gamma_{flat}}_{R}\left( 1+{\Gamma_{iso\_\Delta}}_{R}\frac{K_{h}}{K_{cl}}\left( \frac{e_{cl}}{B_{safe}+H_{stg}} \right) \right)$$

(20)

For anisotropic aquifers the clogging layer correction, is differentiated between left and right as follows:

$${\Gamma_{Q}}_{L}=\frac{{\Gamma_{anis-\Delta_{G}}}_{L}}{1+ {\Gamma_{anis-\Delta_{G}}}_{L}\frac{K_{h}}{K_{cl}}\left( \frac{{\Gamma_{anis-\Delta_{G}}}_{L}\left( H_{stream}-h_{L} \right)+{\Gamma_{anis-\Delta_{G}}}_{R}\left( H_{stream}-h_{R} \right)}{{\Gamma_{anis-\Delta_{G}}}_{L}\left( H_{stream}-h_{L} \right)} \right)\frac{e_{cl}}{2\left( B_{safe}+H_{stg} \right)}}$$

(21)

$${\Gamma_{Q}}_{R}=\frac{{\Gamma_{anis-\Delta_{G}}}_{R}}{1+ {\Gamma_{anis-\Delta_{G}}}_{R}\frac{K_{h}}{K_{cl}}\left( \frac{{\Gamma_{anis-\Delta_{G}}}_{L}\left( H_{stream}-h_{L} \right)+{\Gamma_{anis-\Delta_{G}}}_{R}\left( H_{stream}-h_{R} \right)}{{\Gamma_{anis-\Delta_{G}}}_{R}\left( H_{stream}-h_{R} \right)} \right)\frac{e_{cl}}{2\left( B_{safe}+H_{stg} \right)}}$$

(22)

Text S3

To verify the numerical implementation of Stream Aquifer Flow Exchange (SAFE) method within IWFM, we constructed a hypothetical one-dimensional groundwater problem for which an analytical solution is available. The modeling domain consists of a homogeneous, isotropic, unconfined aquifer with hydraulic conductivity equal to 8.64 m/day, represented by one layer with length of 1000 m. A constant-head boundary of 5.0 m is assigned at the right end of the domain (x=1000 m), while a stream is located at the opposite end (x=0 m). Groundwater exchange with the stream occurs through a streambed layer with hydraulic conductivity of 0.0144 m d⁻¹ and thickness of 1.0 m. IWFM is designed primarily for real-world applications and there is no native one dimensional modeling option. Therefore, we constructed a two dimensional model domain with 1000 m length and 500m width discretized into 10 columns and 5 rows of 100mx100m elements (Figure S7). In IWFM the stream stage cannot be assigned by the user as it is calculated iteratively by the non linear streamflow solver. Therefore, stream stage was controlled indirectly by varying the inflow rate specified at the upstream end of the stream (top left corner of the grid). A series of simulations was performed by varying the inflow rate and therefore the stream stage. Here we focus on two cases: i) a low-stage case with a stream stage of approximately 0.3 m and a high-stage case with a stream stage of approximately 2.0 m

Under the assumptions of steady-state one-dimensional unconfined flow and the Dupuit approximation, groundwater discharge per unit stream length is constant and can be expressed as:

$$q_{gw}=\frac{K}{2L}\left( h_{L}^{2}-h_{o}^{2} \right)$$

(23)

Where positive values indicate flow from the stream to the aquifer and negative values indicate groundwater discharge to the stream. In eq 23 $K$ is the aquifer hydraulic conductivity, $L$ is the distance from the stream to the constant head boundary $h_{L}$, and $h_{o}$ is the groundwater head adjacent to the stream.

The stream aquifer exchange through the streambed is expressed as:

$$q_{s}=C\left( h_{s}-h_{o} \right)$$

(24)

Where $C$ is the stream aquifer conductance and $h_{s}$ is the stream stage

Combining equations (23) and (24) ($q_{gw}= q_{s})$ yields a quadratic equation for $h_{o}$. Once $h_{o}$ is determined, the groundwater head distribution within the aquifer is obtained from:

$$h(x)=\sqrt{h_{L}^{2}+\frac{2q}{K}\left( L-x \right)}$$

(25)

which provides the analytical groundwater profile for comparison with the numerical solution.

Unlike the traditional First Order Exchange (FOE) formulation, which assumes a constant streambed conductance, the SAFE formulation computes an effective conductance that varies dynamically with stream stage and aquifer conditions. SAFE calculates a dimensionless conductance term that accounts for stream penetration, wetted perimeter, aquifer geometry, anisotropy, and streambed clogging effects. As a result, the effective conductance increases with stream stage. Direct comparison of SAFE results with the analytical solution using the assigned streambed conductance is therefore only valid at low stages where the effective SAFE conductance remains close to the prescribed streambed conductance. For higher stages, the analytical solution must use the effective conductance computed by SAFE rather than the nominal streambed conductance.

Figure S8 compares numerical and analytical groundwater head profiles along the bottom row (y=0) of the modeling domain for a low-stage and a high-stage case. For the low-stage case ($h_{s}\approx$ 0.3 m), the effective SAFE conductance is approximately equal to the assigned streambed conductance ($C$ = 0.0144 m/day), which results in excellent agreement between the numerical and analytical solutions (blue lines). For the high-stage case ($h_{s}\approx$ 2.0 m), the effective SAFE conductance increased to approximately 0.056 m/day, which is several times larger than the assigned streambed conductance. When the assigned conductance was used in the analytical solution (orange dashed line), significant discrepancies in both groundwater heads and exchange fluxes are observed relative to the numerical SAFE solution (orange dots). However, when the analytical solution used the effective conductance calculated internally by SAFE (solid orange line), the analytical and numerical groundwater profiles were again in close agreement.

These results demonstrate that the numerical implementation reproduces the expected analytical behavior and confirm that the stage-dependent conductance formulation implemented in SAFE is functioning as intended.

Figure S9 show the effective stream aquifer conductance computed by SAFE as function of stream stage. The results demonstrate a systematic increase in conductance with increasing stage, confirming that stream stage influences stream–aquifer exchange not only through the hydraulic gradient but also through changes in the effective hydraulic connection between the stream and the aquifer. This behavior is a fundamental characteristic of the SAFE formulation and explains the differences observed between the constant-conductance analytical solution and the numerical SAFE simulations at higher stream stages. with stream stage obtained from the SAFE simulations.

Figure S1. Illustration of flow resistance between a stream cross section and an aquifer cross section at the “far enough” distance where the flow is horizontal.

Figure S2. Estimation of “far-enough” distance in the finite element implementation. The blue lines are the edges of the finite elements and the red lines represent streams. The orange lines show the $\boldsymbol{G}_{\boldsymbol{SAFE}}$ distance for the two nodes of the stream segment. The hydraulic head is interpolated along the line that connects the two points at $\boldsymbol{G}_{\boldsymbol{SAFE}}$ distance from the stream nodes (black dots)

Figure S3. Cumulative stream aquifer volume aggregated by reach.

Figure S4. Kernel density estimates (KDEs) of the absolute differences between left and right bank stream aquifer exchange across all stream nodes and time steps. Each curve represents the distribution of difference (in thousands cubic meter per month) for a single time step. The results indicate that over the simulation period most stream nodes exhibit differences in exchange magnitude on the order of 10**^2^** **to** 10^5^ m**^3^**/month corresponding approximately to 500 - 100,000 cubic meter per month

Figure S5. Spatial distribution of average deep percolation for the period 2000-2015 in the San Joaquin River region. Upstream areas on along the right bank receive substantially higher recharge (approximately 200-600 mm/year), whereas downstream recharge decreases to approximately 50 mm/year.

Figure S6. Streambed conductance and hydraulic conductivity profiles along three selected rivers. The corresponding stream aquifer exchange behavior is discussed in the main text and presented in Figure 7

Figure S7. Numerical grid used for the verification example. Because IWFM does not provide a one-dimensional simulation option, the analytical 1D problem was represented using a two-dimensional model domain measuring 1000 m × 500 m and discretized into 10 columns and 5 rows of 100 m × 100 m elements. A stream is located along the left boundary and a constant-head boundary is assigned along the right boundary. Stream inflow is introduced near the upper-left corner of the model domain (y = 500 m). To minimize the local influence of the inflow point on the comparison with the one-dimensional analytical solution, groundwater heads from the row farthest from the inflow location (y = 0 m) were used for the head-profile comparison.

Figure S8. Comparison of analytical and numerical groundwater head profiles for a low-stage case ($h_{s}\approx$ 0.3 m; blue) and a high-stage case ($h_{s}\approx$ 2.0 m; orange). Dots represent numerical SAFE results, dashed lines represent the analytical solution using the assigned streambed conductance ($C$ = 0.0144 m/day), and solid lines represent the analytical solution using the effective conductance calculated internally by SAFE. For the low-stage case, the effective SAFE conductance is nearly identical to the assigned conductance, resulting in excellent agreement between the analytical and numerical solutions. For the high-stage case, SAFE computes a substantially larger effective conductance, and agreement is achieved only when this effective conductance is used in the analytical solution.

Figure S9. Effective stream–aquifer conductance calculated by SAFE as a function of stream stage. The dashed horizontal line indicates the assigned streambed conductance (0.0144 m/day). At low stream stages, the effective SAFE conductance is very close to the assigned streambed conductance. As stream stage increases, SAFE predicts progressively larger conductance values due to the dependence of stream–aquifer connectivity on wetted perimeter, penetration depth, aquifer geometry, anisotropy, and streambed clogging effects.

Table S1. Parameters of riverbed materials. h_ce_ is the entry pressure of the aquifer material and K is the hydraulic conductivity.

| Soil type | h_ce_ (cm) | K (m/day) |
| --- | --- | --- |
| Sand | 15.98 | 5.04 |
| Loamy sand | 20.58 | 1.4664 |
| Sandy loam | 30.2 | 0.6216 |
| Loam | 40.12 | 0.1632 |
| Silty loam | 50.87 | 0.3168 |
| Sand clay loam | 59.61 | 0.1032 |
| Clay loam | 56.43 | 0.0552 |
| Silty clay loam | 70.33 | 0.036 |
| Sandy clay | 79.48 | 0.0288 |
| Silty clay | 76.54 | 0.0216 |
| Clay | 85.6 | 0.0144 |

Table S2. Flat isotropic conductance parameters.

| Range of $W_{p}^{N}$ | Range of $d_{p}^{N}$ | $a_{1}$ | $a_{2}$ |
| --- | --- | --- | --- |
| $W_{p}^{N}\leq1.0$ | $d_{p}^{N}\leq0.2$ | 0.890 | -2.430 |
| $W_{p}^{N}\leq1.0$ | $0.2\leq d_{p}^{N}\leq0.5$ | 0.538 | -0.387 |
| ${1.0\leq W}_{p}^{N}\leq3.0$ | $d_{p}^{N}\leq0.2$ | 0.819 | -1.340 |
| ${1.0\leq W}_{p}^{N}\leq3.0$ | $0.2\leq d_{p}^{N}\leq0.5$ | 0.672 | -0.542 |
| ${1.0\leq W}_{p}^{N}\leq3.0$ | $0.5\leq d_{p}^{N}\leq0.9$ | 0.567 | -0.330 |

References

Dogrul, E.C., & Kadir, T.N. (2023). DWR technical memorandum: theoretical documentation for the Integrated Water Flow Model (IWFM v4.0-2015), revision 1443: Theoretical documentation. Sacramento, (CA): Integrated Hydrological Models Development Unit, Modeling Support Branch, Bay Delta Modeling Support Office, California Department of Water Resources. https://data.cnra.ca.gov/dataset/5c4b82c9-d219-4d71-a6cc-7ea6ccbaa54b/resource/311462d8-6cb5-4259-bd2c-c1e36a5475be/download/iwfm-2015.2.1443.zip

Morel-Seytoux, H. J. (2009). The turning factor in the estimation of stream-aquifer seepage. Ground Water, 47(2), 205–212. <https://doi.org/10.1111/j.1745-6584.2008.00512.x>

Morel-Seytoux, H. J., Mehl, S., & Morgado, K. (2014). Factors influencing the stream-aquifer flow exchange coefficient. Groundwater, 52(5), 775–781. <https://doi.org/10.1111/gwat.12112>

Morel-Seytoux, H. J., Miller, C. D., Mehl, S., & Miracapillo, C. (2018). Achilles’ heel of integrated hydrologic models: The stream-aquifer flow exchange, and proposed alternative. Journal of Hydrology, 564, 900–908. https://doi.org/10.1016/j.jhydrol.2018.07.010
